# 3D bioprinting of hepatocytes: core–shell structured co-cultures with fibroblasts for enhanced functionality

**DOI:** 10.1038/s41598-021-84384-6

**Published:** 2021-03-04

**Authors:** Rania Taymour, David Kilian, Tilman Ahlfeld, Michael Gelinsky, Anja Lode

**Affiliations:** Centre for Translational Bone, Joint and Soft Tissue Research, Faculty of Medicine and University Hospital Carl Gustav Carus, TU Dresden, Dresden, Germany

**Keywords:** Biological models, Biomaterials - cells, Biomedical engineering, Biomaterials

## Abstract

With the aim of understanding and recapitulating cellular interactions of hepatocytes in their physiological microenvironment and to generate an artificial 3D in vitro model, a co-culture system using 3D extrusion bioprinting was developed. A bioink based on alginate and methylcellulose (algMC) was first shown to be suitable for bioprinting of hepatocytes; the addition of Matrigel to algMC enhanced proliferation and morphology of them in monophasic scaffolds. Towards a more complex system that allows studying cellular interactions, we applied core–shell bioprinting to establish tailored 3D co-culture models for hepatocytes. The bioinks were specifically functionalized with natural matrix components (based on human plasma, fibrin or Matrigel) and used to co-print fibroblasts and hepatocytes in a spatially defined, coaxial manner. Fibroblasts acted as supportive cells for co-cultured hepatocytes, stimulating the expression of certain biomarkers of hepatocytes like albumin. Furthermore, matrix functionalization positively influenced both cell types in their respective compartments by enhancing their adhesion, viability, proliferation and function. In conclusion, we established a functional co-culture model with independently tunable compartments for different cell types via core–shell bioprinting. This provides the basis for more complex in vitro models allowing co-cultivation of hepatocytes with other liver-specific cell types to closely resemble the liver microenvironment.

## Introduction

The concepts of liver tissue engineering aim to mimic not only the tissue specific composition of the extracellular matrix (ECM) but also the (micro) architecture of the organ in order to generate functional constructs providing for example in vitro drug screening/toxicity testing platforms or disease models*.* The liver is a particularly complex organ composed of lobules as building units, with each lobule composed of four tissue systems: parenchymal (hepatocytes) and non-parenchymal cells (e.g. epithelial and endothelial cells), an intrahepatic vascular system as well as bile ducts and interconnected channels. Therefore, in order to realize bioengineered 3D liver constructs, the two most important factors for cells would be a supportive biomaterial and their tissue-like patterning^1^.

In current research, the preservation of long-term functionality of hepatocytes within tissue engineered constructs is one of the major challenges. Recent approaches in the field focused on the development of biomaterial-mediated systems which provide specific biochemical and topological cues: The translation of cell cultures from 2D on plastic plates, which provides primary insights into cellular behavior and interaction, to 3D micro-patterned co-cultures of several cell types resulting in a closer resemblance of the physiological microenvironment^2–4^. However, there is a need for developing novel strategies towards fine-tuning the spatial arrangement of cells and microenvironmental factors as well as for the integration of vascular and biliary channels as critical components for liver function^2^. To bridge this gap, modern technologies such as 3D bioprinting offer great potential to realize multiscale tissue engineering by combining the micro- and macroscale level, which is essential for liver reconstruction^5^. 3D bioprinting is a powerful tool enabling the fabrication of highly organized cell-laden constructs, utilizing various biomaterials, bioactive molecules and different cell types, arranged in a spatially defined pattern. One common type of 3D bioprinting, especially used in this study, is extrusion-based bioprinting (also called bioplotting) suitable to generate volumetric tissue constructs^6,7^ comprising encapsulated cells in extrudable inks. The so-called bioinks are strandwise deposited in layer-by-layer fashion according to a respective design to build 3D constructs with defined architecture; after printing, the constructs are stabilized by crosslinking of the ink^8,9^. Based on previous approaches considering stabilization of low viscosity bioinks or formation of tubular structures^10,11^, bioprinting in a core–shell fashion can also be a promising option for the spatially defined arrangement of several cell types: Two (or even more) bioinks can be simultaneously extruded through coaxial needles forming strands with two discrete compartments, the inner core which is completely enclosed within the outer (potentially stabilizing) shell^12^. Thus, this technique enables in principle printing of different cell types in close proximity, allowing their interaction. Moreover, channel-like structures can be easily integrated in tissue engineering constructs, resembling natural tubular systems like vasculature^13^.

When choosing an ink for bioprinting, a number of properties should be considered such as viscoelasticity and shear thinning behavior for printing with high shape fidelity, cell-compatible composition and gelation mechanism, and ideally cell-supportive biochemical and structural features^14^. Alginate is a widely used biomaterial for cell encapsulation due to its favorable physical properties and biocompatibility^15^. However, the use of alginate in cytocompatible concentrations for extrusion-based bioprinting is strongly limited by its low viscosity and therefore, various strategies have been developed to make it applicable for printing of volumetric constructs^6,16^. One strategy is the internal stabilization via blending with methylcellulose, a biocompatible biopolymer, which temporarily increases the viscosity for printing, thus improving the shear thinning behavior of the bioink. After printing and ionic crosslinking of the alginate, this temporary thickener diffuses to a large extent from the 3D alginate network over time^17,18^. Previously, we have shown that a blend consisting of 3% alginate and 9% methylcellulose (algMC) is suitable for printing volumetric constructs with excellent shape fidelity that are stable post-printing and post-cross-linking while ensuring survival, maintaining metabolic activity and allowing differentiation of the various encapsulated cell types^18–21^.

Alginate is known to lack cell binding sites, thus most cell types remain embedded in an unphysiological state with rounded morphology, resulting in suboptimal viability and function^22^. Therefore, with the aim of modifying the cell microenvironment to provide binding sites for cell attachment, multiple strategies have been employed: Covalently modifying alginate with an RGD-peptide sequence^23,24^ or incorporation of extracellular matrix (ECM) components like collagen, fibrin and laminin^25,26^ have shown to support cell adhesion, proliferation and differentiation. Matrigel, a gelatinous ECM-like protein mixture, has been also used in combination with alginate to provide essential growth factors and ECM proteins for a more bioactive microenvironment^27,28^. Very recently, our group has reported on the functionalization of the algMC ink with human blood plasma which is also rich in growth factors, cytokines and structural ECM components: the study showed how this functionalized ink could enhance attachment and intercellular interactions and therefore improve viability and cell function^21^.

For a functional 3D liver model, cell–cell interactions either homotypic or heterotypic are particularly crucial for regulating proliferation and differentiation of liver cells^29^. With the intention to mimic the physiological microenvironment, co-cultures of hepatocytes with non-parenchymal cells were established to investigate the hypothesis that these cellular interactions, based on signaling pathways of soluble molecules or factors^30^, enhance the potential of the hepatocytes survival and function^31^. One commonly used cell type for co-culture studies with hepatocytes is the fibroblast; these cells can be applied in indirect or direct contact co-cultures for understanding the effects of these interactions on the phenotype of hepatocytes. In both co-culture types, a supportive effect of the fibroblasts on hepatocyte survival and function was observed^30,32,33^.

The aim of the present study was to establish a 3D core–shell bioprinting-based concept for the biofabrication of liver models using algMC as the base bioink to provide stable core and shell compartments supporting encapsulated cells in each phase. Using HepG2, a carcinoma-derived immortalized liver cell line as a model system, we developed an optimized ink in order to better recapitulate their respective biochemical microenvironment and, therefore, support cellular function. This was achieved by functionalizing our previously developed algMC blend^18^ with Matrigel. The applicability of the developed ink and core–shell printing in combination with HepG2 cells towards 3D liver models was evaluated. As a further step in the direction of tissue complexity, a fibroblasts-HepG2 co-culture system was established by using coaxial extrusion. After cultivating the printed core–shell constructs for several weeks, cell–cell interactions were studied through evaluation of cellular morphology and distribution as well as expression of relevant biomarker proteins to evaluate the suitability of the developed 3D model to study the influence of the microenvironment on the phenotype and performance of hepatocytes.

## Materials and methods

### Bioink preparation

The basic ink consisting of a blend of 3 wt% alginate and 9 wt% methylcellulose (algMC) was prepared according to the protocol described previously^18^. In brief, PRONOVA UP LVM sodium alginate, Viscosity [mPa*s]: 20–200, G/M Ratio: ≤ 1, (Novamatrix, Norway) was dissolved at a concentration of 30 mg ml^−1^ in Hank’s Balanced Salt Solution (HBSS) by stirring overnight. Then, the solution was autoclaved for sterilization (121 °C for 20 min in a Systec D-23 table-top autoclave, Germany), and 9 wt% of autoclaved methylcellulose powder (4000 cP, Sigma Aldrich, USA) was added and allowed to swell for 2 h. For Matrigel supplementation of the algMC blend, 5 vol/wt% Matrigel (Corning Matrigel membrane matrix; Fisher Scientific, Germany) was added to the blend after MC swelling and immediately before adding cells. For preparation of the fibrin-supplemented ink, 20 mg ml^−1^ fibrinogen from Tisseel (Fibrin Sealant) kit (Baxter, USA) was added to 3 wt% autoclaved alginate solution before adding 9 wt% MC powder. The plasma-suplemented ink was prepared, as recently described^21^, by dissolving 30 mg ml^−1^ alginic acid sodium salt in thawed human plasma (Fresh frozen human blood plasma was provided by the local blood bank (German Red Cross, Dresden, Germany)) before 9 wt% MC powder was added and allowed to swell. After preparing the blends, cells were prepared as follows and added to the inks to produce the bioinks.

*Cells:* Human hepatocellular carcinoma (HepG2) and mouse NIH 3T3 fibroblast cell lines were used as models and purchased from DSMZ—German Collection of Microorganisms and Cell Cultures (Braunschweig, Germany). Both cell lines were expanded in cell culture medium consisting of Dulbecco's modified Eagle's medium (DMEM; Gibco, Life Technologies, Germany) with 10 vol% fetal calf serum (FCS; Corning, USA) and 100 U ml^−1^ penicillin and 100 µg ml^−1^ streptomycin (P/S; Biochrom, Germany) at 37 °C in a humidified 5% CO_2_ atmosphere. To label the cells, HepG2 cell suspension was incubated at 37 °C in a 5% CO_2_ incubator for 15 min with DiD (Invitrogen Vybrant DiD Cell Labeling Solution; ThermoFisher, USA) while NIH 3T3 were incubated under same conditions with DiI (Invitrogen Vybrant DiI Cell Labeling Solution; ThermoFisher). After harvesting and optional labeling, 5 × 10^6^ cells were resuspended in 100 μl of DMEM with 10% FCS and then used to prepare the bioinks by gently mixing in the 100 μl cell suspension into 1 g ink.

### 3D bioprinting of scaffolds: monophasic and core–shell strand plotting

In this work, the extrusion-based technique of 3D plotting was used^10,18^. Scaffolds were fabricated using pneumatic BioScaffolder 3.1 from GeSiM (Radeberg, Germany) under sterile conditions. The pasty bioinks were dispensed through conical dosing needles (Nordson EFD, Germany) using compressed air; strands were deposited in a layer-by-layer fashion, with parallel alignment in each layer and a shift in orientation of 90° between the layers, in 12 well-plates using air as plotting medium.

*Monophasic scaffolds:* For monoculture experiments, HepG2-laden hydrogel blends (algMC with or without Matrigel) were extruded through needles with an outlet diameter of 410 μm, forming uniform strands, with a dosing pressure of 60 kPa and a printing speed of 8 mm s^−1^. The dimensions of square shaped scaffolds consisting of 4 layers (strand distance ca. 1.4 mm, total height ca. 1.3 mm) were 8 mm × 8 mm. After plotting, scaffolds were immediately crosslinked for 10 min in 100 mM CaCl_2_ solution before being transferred to cell culture medium for cultivation.

*Core–shell scaffolds:* For co-culture experiments, coaxial plotting was employed, in which the print head was designed with two concentric needles (GeSiM, Radeberg, Germany) attached to core and shell ink cartridges and connected to independently controllable compressed air valves for extruding HepG2-laden bioink as shell and NIH 3T3-laden bioink as core within one strand. Scaffolds were printed with the dimensions of 8 mm × 8 mm forming 2 layers. For the shell bioink (HepG2 in algMC + Matrigel), a needle with an outlet diameter of 800 µm and a dosing pressure of 70 kPa was used. For the core bioinks (NIH 3T3 in either algMC, algMC + fibrin or algMC + plasma), needles with an outlet diameter of 400 µm and dosing pressures of 70 kPa, 60 kPa and 50 kPa, respectively, were used. After plotting, scaffolds were stabilized by crosslinking in 100 mM CaCl_2_ for 10 min; for scaffolds containing fibrin in the core, thrombin from Tisseel (Fibrin Sealant) kit (Baxter, USA) was added to the 100 mM CaCl_2_ solution in a concentration of 20 U ml^−1^. All scaffolds were finally washed in HBSS for 10 min to get rid of excess crosslinking solution before being cultured in cell culture medium.

### Cell viability, morphology and metabolic activity within bioprinted constructs

*Viability:* Cell viability was assessed by simultaneous fluorescence staining of live and dead cells. At given time points of cultivation, three cell-laden scaffolds per type were incubated in culture medium containing Calcein AM and Ethidium homodimer-1 (Invitrogen) for 20 min under cell culture conditions. After staining, *z*-stack images were acquired using a Leica TCS SP5 confocal laser scanning microscope (cLSM; Leica, Germany). For quantification of live and dead cells, Imaris software 9.5 (Oxford Instruments) was used to calculate the volume ratio of cell clusters formed over the culture period.

*Morphology*: To observe the morphology and organization of the cytoskeleton, cell-laden scaffolds were taken at given time points, fixed in 4% formaldehyde, permeabilised by 1% Triton X100, blocked with 3% bovine serum albumin (BSA, Albumin Fraction V, Roth, Germany). Thereafter, cell nuclei were stained with 1 µg ml^−1^ DAPI (Gibco life technologies, USA), while the cytoskeleton of the cells was stained with Phalloidin-iFluor 488 Reagent (Abcam, USA). After staining, *z*-stack images were acquired using a Leica TCS SP5 cLSM.

*Metabolic activity:* Cell metabolic activity was assessed after 7 days of culture using 3-(4,5-dimethylthiazol-2-yl)-2,5-diphenyltetrazolium bromide (MTT, Sigma-Aldrich, USA) which is converted by mitochondrial dehydrogenases of living cells to water-insoluble formazan of violet color. Core–shell scaffolds with cells encapsulated either in core or shell were incubated for 2–4 h in cell culture medium containing 500 μg ml^−1^ of MTT under cell culture conditions and imaged using a stereo light microscope (M205 C equipped with DFC295 camera, Leica, Germany).

### Cell proliferation within bioprinted constructs

*DNA assay:* Cell number in the hydrogel scaffolds over the cultivation time was assessed by quantifying the DNA content of the scaffolds which can be correlated with the cell number using a calibration line. For this, three scaffolds per type were collected at given time points and frozen at − 80 °C. After completion of the culture period of 10 days, all scaffolds were thawed, 100 mM sodium citrate was added and the scaffolds were incubated overnight at 60 °C to dissolve the hydrogel and to lyse all the encapsulated cells and release the DNA. DNA content was quantified by adding QuantiFluor (dsDNA Assay, Promega, USA) to the cell lysate and measuring fluorescence signal after reaction at 485/535 nm using a microplate reader (infinite M200pro, Tecan, Switzerland).

*EdU staining:* To detect newly formed cells, Click-iT EdU imaging kit (ThermoFisher Scientific, Germany) was used. At given time points, scaffolds were incubated for 4 h with EdU working solution, prepared in cell culture medium according to manufacturer’s protocol, under cell culture conditions. Thereafter, scaffolds were fixed with 4% formaldehyde, washed in HBSS and permeabilized with 0.5% Triton X100 for 1 h. Detection of EdU was performed by incubating the scaffolds with Click-iT reaction cocktail prepared according to manufacturer’s protocol. Finally, DAPI was used for nuclear staining before imaging by Leica TCS SP5 cLSM.

### Functionality assay

In order to quantify albumin secretion, the cell culture supernatant from HepG2-laden scaffolds was collected at indicated time points and stored at − 80 °C until analysis. Albumin secreted by the cells was determined by detection in this cell culture medium using a human albumin ELISA Kit (Merck, Sigma Aldrich, Darmstadt, Germany) following the manufacturer’s instructions. Optical density at 450 nm was measured with a microplate reader (infinite M200pro, Tecan). Experiments were performed in triplicate.

### Immunofluorescence analysis of bioprinted constructs

In order to observe specific markers and morphological changes of the embedded cells in scaffolds, immunofluorescence staining was performed. At given time points, cell-laden scaffolds were collected, fixed with 4% formaldehyde, washed in HBSS, permeabilized with 1% Triton X100 and then blocked with 3% bovine serum albumin (BSA). For specific biomarker staining, different antibodies were applied: mouse *Anti-Human Serum Albumin* antibody (ab10241; Abcam, Germany, dilution 1:2000) labeled with AlexaFluor 568-tagged goat anti-mouse secondary antibody (ab175473; Abcam), mouse *Anti-Human Cytokeratin 19* monoclonal antibody (ThermoFisher scientific, Germany, dilution 1:500) detected via AlexaFluor 568-tagged goat anti-mouse secondary antibody, rabbit *Anti-Human α1 antitrypsin* polyclonal antibody (ThermoFisher scientific, Germany, dilution 1:250) detected via AlexaFlour 488 goat anti-rabbit secondary antibody (ThermoFisher scientific). Cell nuclei were stained with DAPI and cytoskeletons with Phalloidin-iFluor 488 Reagent. Expression of cell surface markers and morphology was observed with a Leica TCS SP5 cLSM.

### Rheological characterization of the inks

Rheological investigation of the hydrogel blends was performed using a plate rheometer (Rheotest RN 4, Medingen, Germany) with a plate–plate-distance of 0.1 mm. Shear thinning was tested by increasing the shear rate from 0 to 100 s^−1^ over 600 s (increment 0.17 s^−1^).

### Mechanical characterization of plotted and cross-linked constructs

To evaluate the mechanical behavior of the hydrogels after plotting and crosslinking, uniaxial compressive tests were applied to monophasic and core–shell scaffolds with macropores (base 10 × 10 mm^2^, height: 10 layers for monophasic and 4 layers for core–shell scaffolds) at day 1 after incubation in cell culture medium utilizing an universal testing machine (Zwick-Roell Z010 equipped with a 100 N load cell, Zwick, Germany) with a compression velocity of 5 mm min^−1^. Compressive modulus was determined from the obtained stress–strain curves.

### Statistical analysis

GraphPad Prism 8 was used for depiction of data and for statistical analysis. ANOVA with Bonferroni correction was performed to determine statistical significance considering a confidence interval of *p* < 0.05. For experiments comparing between two experimental groups only, unpaired t-test with Welch correction was chosen accordingly.

## Results

### Bioink development for encapsulation of hepatocytes by 3D bioplotting

In order to enhance the 3D microenvironment for encapsulated cells inside the 3D printed algMC network that does not offer biologically active cues or adhesion sites for hepatocytes allowing cell attachment, proliferation or colony formation, Matrigel was added at a concentration of 5% to provide supportive matrix proteins for the encapsulated cells. The influence of Matrigel on hepatocyte viability, morphology and biological function was investigated in a comparative study conducted with HepG2 printed in algMC with and without addition of Matrigel.

#### Viability of HepG2 in algMC printed constructs with and without Matrigel

**Figure 1:**
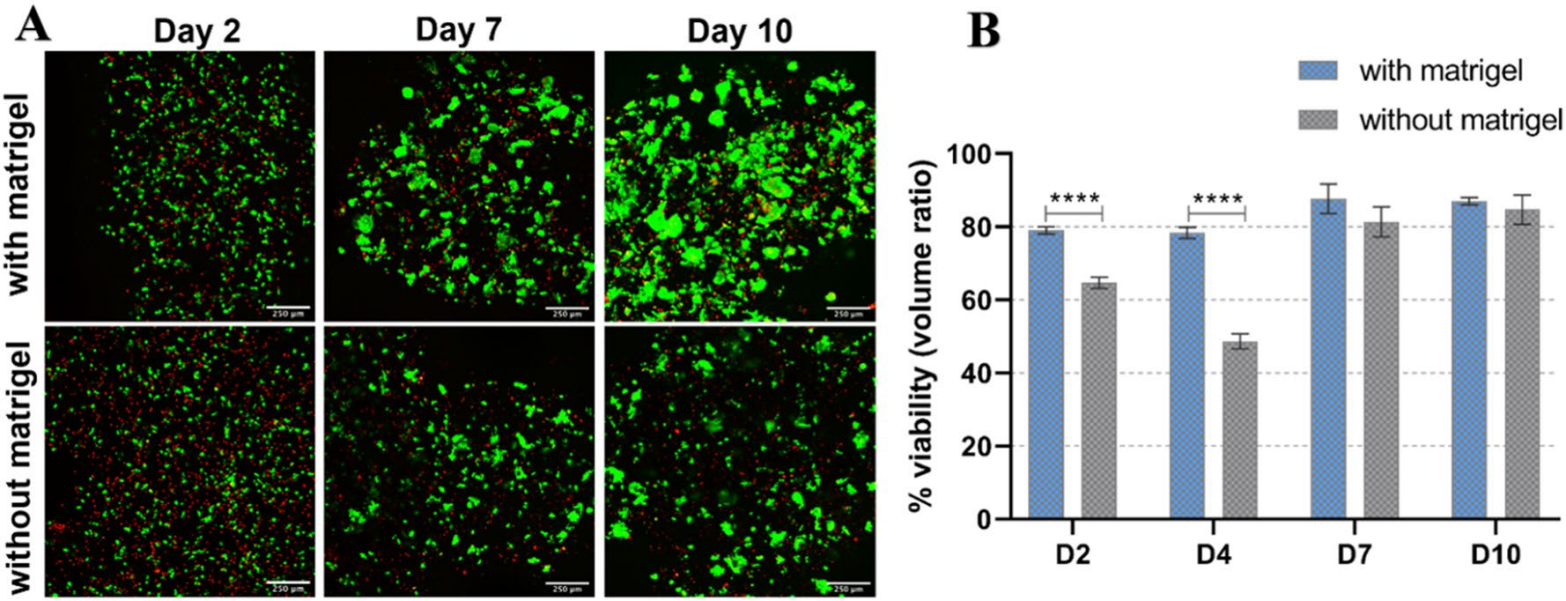
Viability of HepG2 embedded in algMC with and without Matrigel. (**A**) cLSM images of live/dead staining of HepG2 embedded in printed scaffolds at day 2, 7, and 10 of cultivation. Viable cells stained green (calcein), whereas dead cells are stained red (ethidium homodimer-1); scale bar = 250 µm. (**B**) Percent viability (volume ratio of viable vs. total clusters) comparing the two conditions (n = 6, mean ± SD, ****p < 0.0001).

In order to investigate the viability of encapsulated HepG2, bioprinted and crosslinked constructs were cultured for at least 10 days. At different time points of culture, live and dead cells were simultaneously stained in the scaffolds with Calcein AM and EthD-1. Images shown in Fig. 1A reveal that in both conditions and over the whole culture period, majority of the cells survived the printing and cultivation process (stained green). Hepatocytes grew into clusters over time which increased not only in number but also in size, i.e. they integrated with each other to form larger colonies. However, a noticeable difference appeared comparing cells in Matrigel-supplemented to non-supplemented algMC: cell aggregates formed in algMC + Matrigel were larger and more numerous especially on day 7 and day 10 compared to those grown without Matrigel. Since the cells tend to grow in clusters, they could not be analyzed via individual cell counting. Therefore, in this case, viability of the cells was quantified as a function of volume ratio, by determining the volume of live and dead signals. Viability in algMC + Matrigel was generally in the range of 80–90% (Fig. 1B). Whereas on day 2 and 4 a significant difference to viability in unmodified algMC was observed, there was no significant difference on day 7 and day 10 (Fig. 1B). However, live/dead images, especially at day 10, showed more pronounced cluster formation in the presence of Matrigel (Fig. 1A) (for visualization of separate channels with live and dead signals, see Supplementary information Fig. S1).

#### Proliferation of HepG2 in algMC printed constructs with and without Matrigel

**Figure 2:**
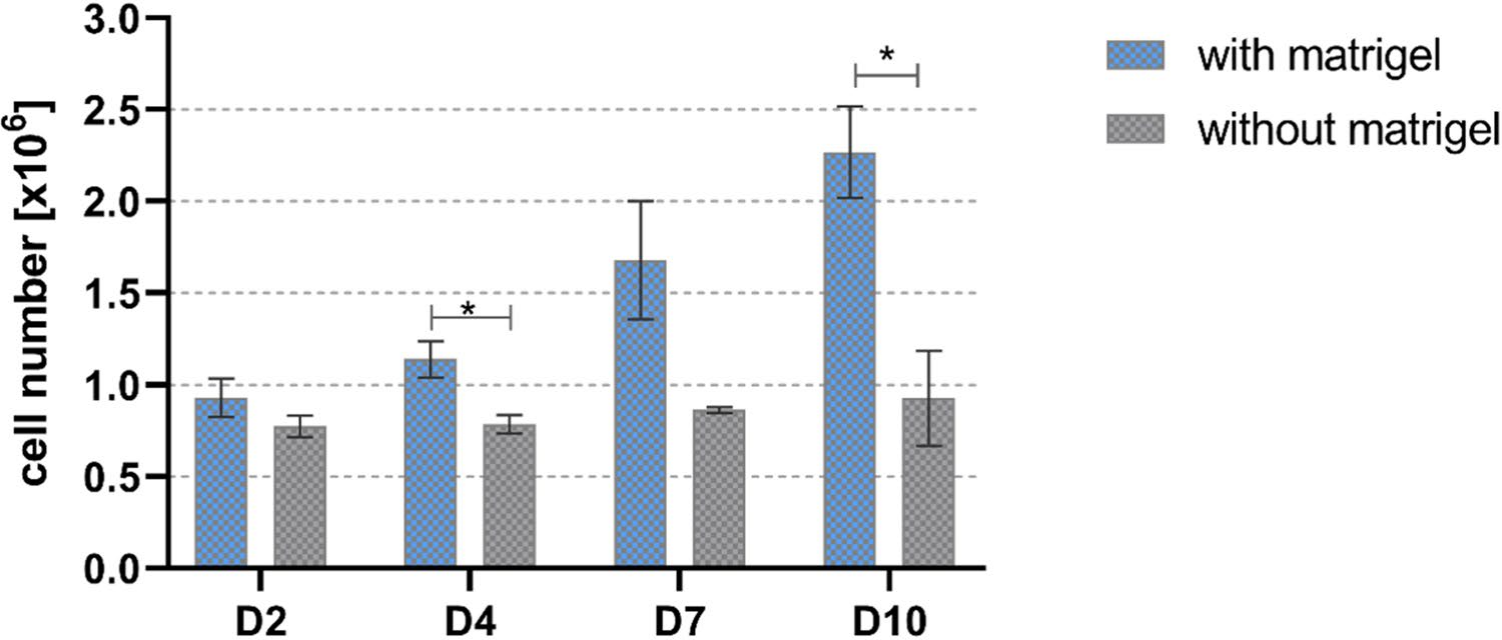
Quantification of HepG2 cell number in printed algMC constructs with and without Matrigel. The DNA content of the scaffolds and therefore corresponding cell number was quantified at different time points (n = 3; mean ± SD; * *p* < 0.05).

Since a higher number of viable cells and bigger cell clusters were visible in Matrigel-supplemented algMC constructs (Fig. 1A), an enhanced proliferation of the cells is suggested. Therefore, DNA assay was performed to quantify the number of cells present in the scaffolds at given time points of cultivation. As shown in Fig. 2, the number of hepatocytes in algMC + Matrigel constructs increased gradually over the period of culture from day 2 to day 10, whereas the hepatocytes in pure algMC constructs showed a constant cell number over time. A significant difference in the cell numbers determined for Matrigel-supplemented vs. Matrigel-free algMC was observed at days 4 and 10 of culture.

**Figure 3:**
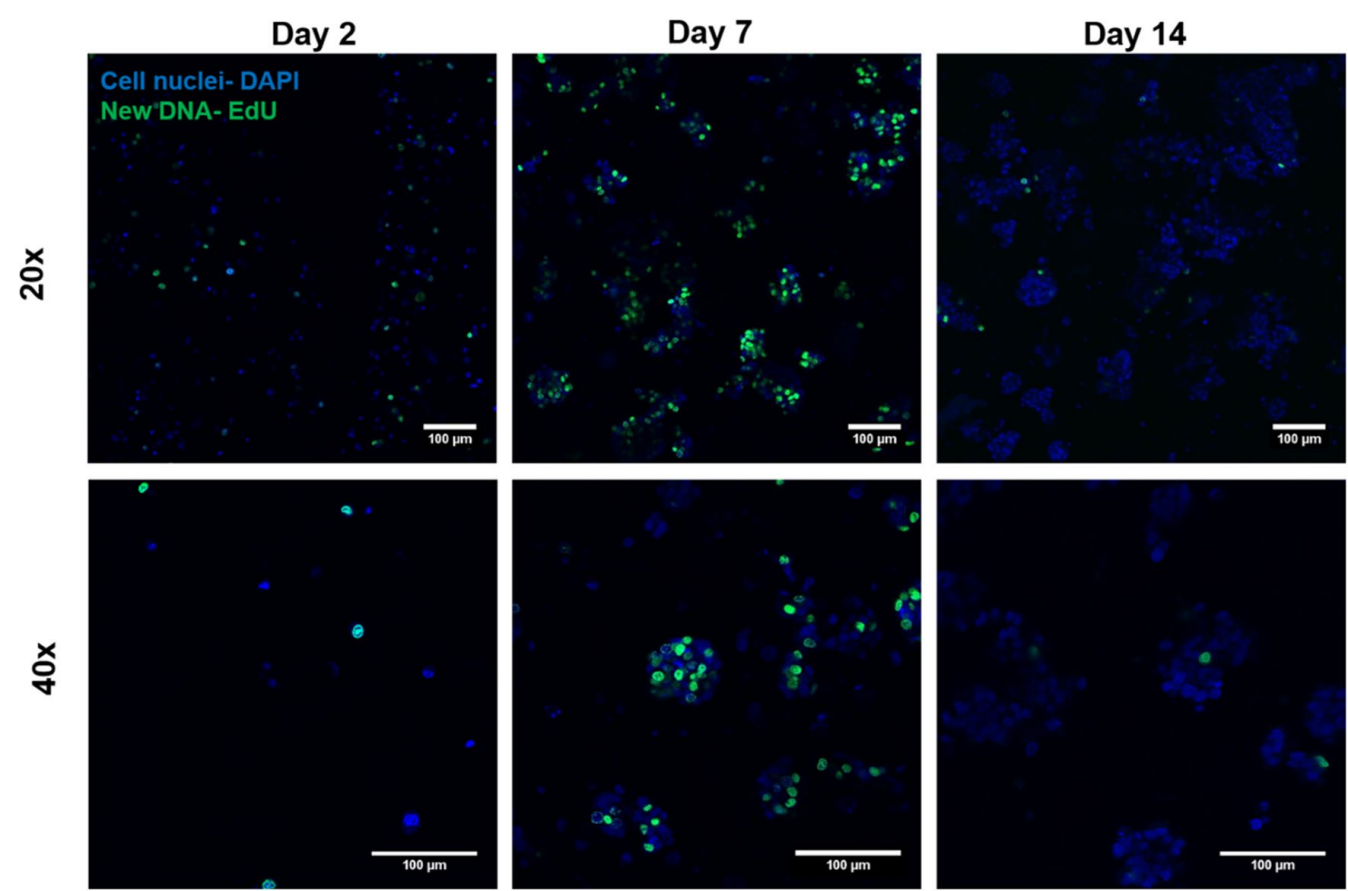
Proliferation of HepG2 embedded in algMC with Matrigel – EdU proliferation assay. Cell nuclei were stained with DAPI (blue); representing original cells, while cells labelled with EdU are shown in green; representing newly formed DNA and therefore mitosis. Upper tile (20 × magnification) shows the distribution of newly formed cells allover a specific region of the scaffold. Lower tile (40 × magnification) shows distribution of cells within the formed clusters and their rate of proliferation; scale bars represent 100 µm.

In order to confirm the results of DNA quantification indicating cell proliferation, EdU assay was performed to stain newly formed DNA during mitosis to recognize the distribution of proliferating cells within clusters formed and to visualize the rate of proliferation. As shown in Fig. 3, cells embedded and bioprinted in algMC + matrigel started to proliferate on day 2 with some newly formed single cells (shown in green). On day 7, a higher number of newly formed cells were observed which are widely distributed within the clusters indicating a higher rate of proliferation. By day 14, only a low number of newly formed cells was observed within the larger clusters suggesting a reduced rate of proliferation at later time points of cultivation. In contrast, there was a lower rate of cell proliferation in unmodified algMC; this was clearly visible on day 7 with less number of clusters and new DNA (Supplementary Fig. S2).

#### Morphology and organization of HepG2 in algMC printed constructs with and without Matrigel

**Figure 4:**
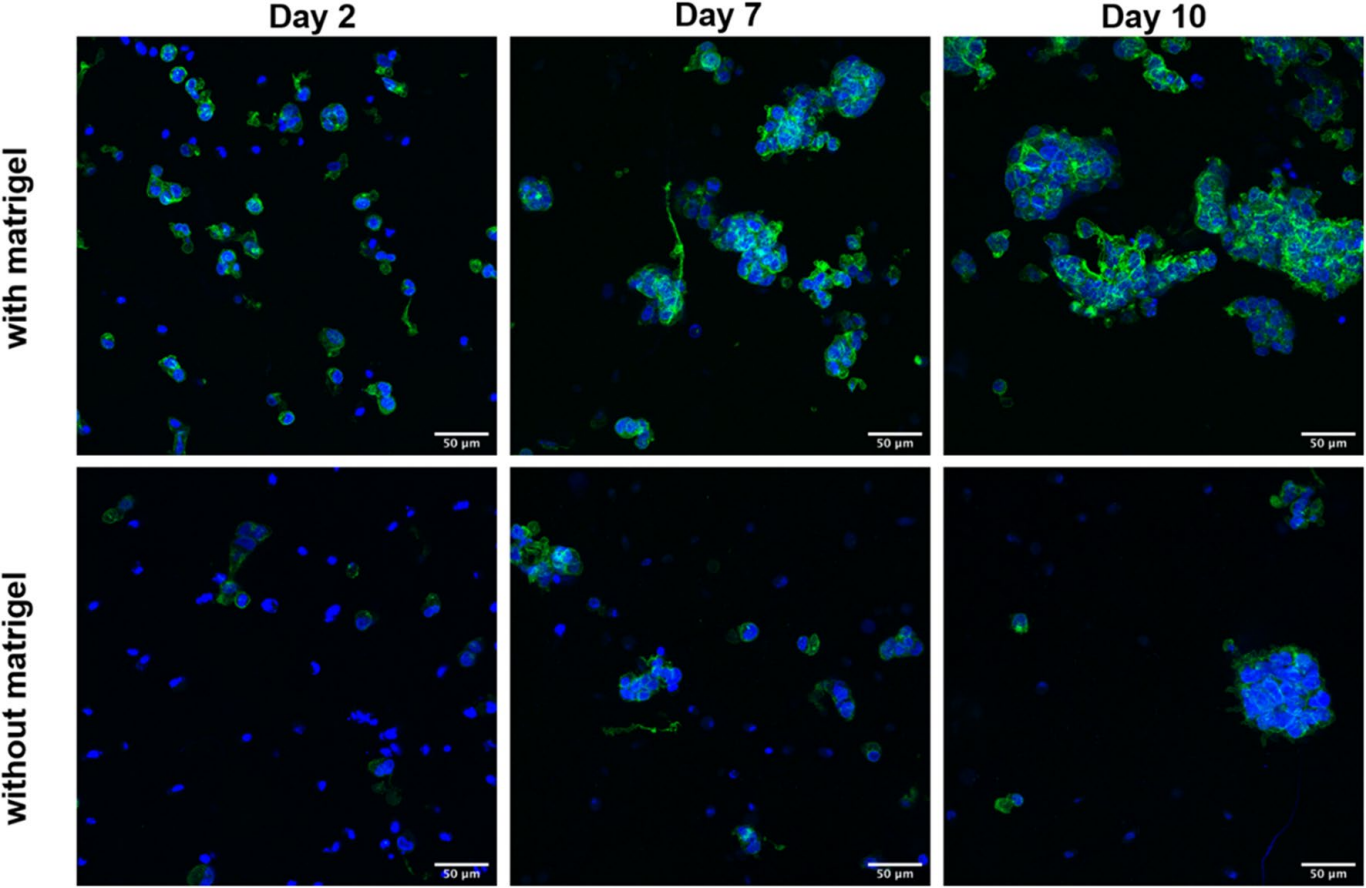
Morphology and organization of HepG2 embedded in algMC with (upper panel) and without Matrigel (lower panel) after 2, 7 and 10 days of cultivation. Cell nuclei are stained in blue (DAPI) while cytoskeletons appear in green (actin staining with Phalloidin-iFluor 488); scale bars represent 50 µm.

For visualization of the morphology and organization of the cells within the bioprinted hydrogel scaffolds, actin cytoskeleton and nuclei were stained at different time points of culture (Fig. 4). Comparing cellular morphology between both conditions, we observed that inside algMC with Matrigel cluster formation started at day 2 and was more pronounced than in algMC without Matrigel. In general, clusters formed in the presence of Matrigel showed more defined and organized architecture of the actin filaments-based network of the cells as well as multicellular aggregates that increased in size and number throughout the culture period (day 7 and day 10) compared to the hydrogel without Matrigel.

#### Functionality of HepG2 in algMC printed constructs with and without Matrigel

**Figure 5:**
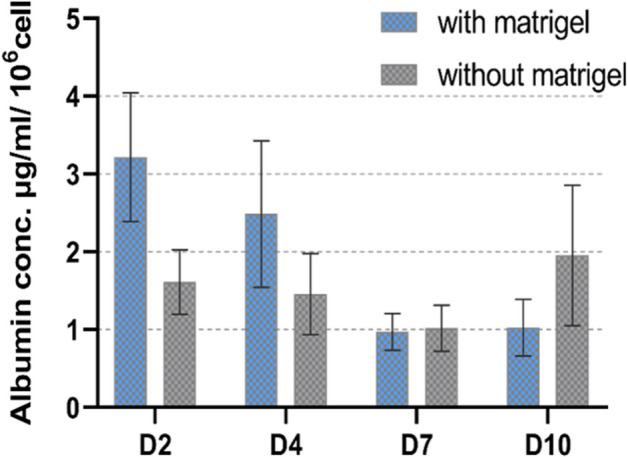
Functional analysis of embedded HepG2 in algMC with and without Matrigel-albumin secretion into cell culture supernatants after 2, 4, 7 and 10 days of cultivation, normalized to the cell number obtained by DNA quantification (n = 6; mean ± SD).

For assessing the functionality of the embedded HepG2, albumin secretion into the supernatant was analyzed. As shown in Fig. 5, at all investigated time points of cultivation, albumin secretion was observed in both conditions, indicating that bioprinted HepG2 are functional in the hydrogels. There was no significant differences observed between secreted albumin in Matrigel-supplemented vs. Matrigel-free scaffolds.

### Spatially defined pattern of indirect hepatocyte-fibroblast co-culture in a core–shell bioprinted system

**Figure 6:**
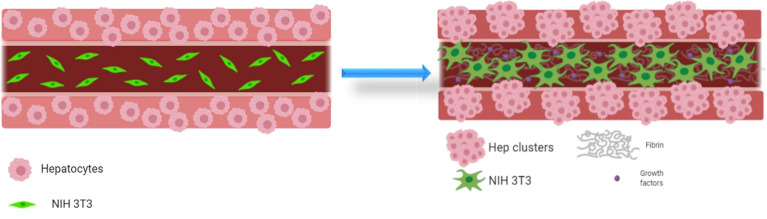
Graphical representation of the hepatocyte-fibroblast co-culture concept, showing hepatocytes encapsulated in the shell are coaxially printed with NIH 3T3 fibroblasts in the core, both co-printed as single cells in their respective bioink (left). An influence of the core composition (with or without fibroblasts and matrix components) on hepatocyte phenotype is hypothesized as they might grow into clusters within their shell compartment and fibroblasts spread to form networks over the cultivation period (right). Images created using Biorender.com software.

After determining a suitable hydrogel material that supports hepatocytes growth and function in a 3D environment, we aimed to investigate their performance in complex co-culture systems fabricated by core–shell 3D bioplotting. HepG2 were encapsulated in the shell compartment consisting of algMC + Matrigel while NIH 3T3 fibroblasts, which act as supporting cells for the hepatocytes, were encapsulated in the core compartment. Hence, the cells were not cultured in direct contact but they were rather expected to communicate through the micro-pores of the encapsulating hydrogel—the effect of this type of co-culture on the behavior of HepG2 was analyzed. Furthermore, in order to investigate the influence of the microenvironment on the fibroblast viability and activity, that is hypothesized to influence in turn the neighboring hepatocytes, NIH 3T3 fibroblasts were encapsulated in different core materials: algMC, algMC + fibrin and algMC + plasma. The functionalization of algMC was thereby expected to enhance fibroblast attachment and spreading and therefore matrix formation (Fig. 6).

#### Fabrication of cell-laden core–shell strand scaffolds

**Figure 7:**
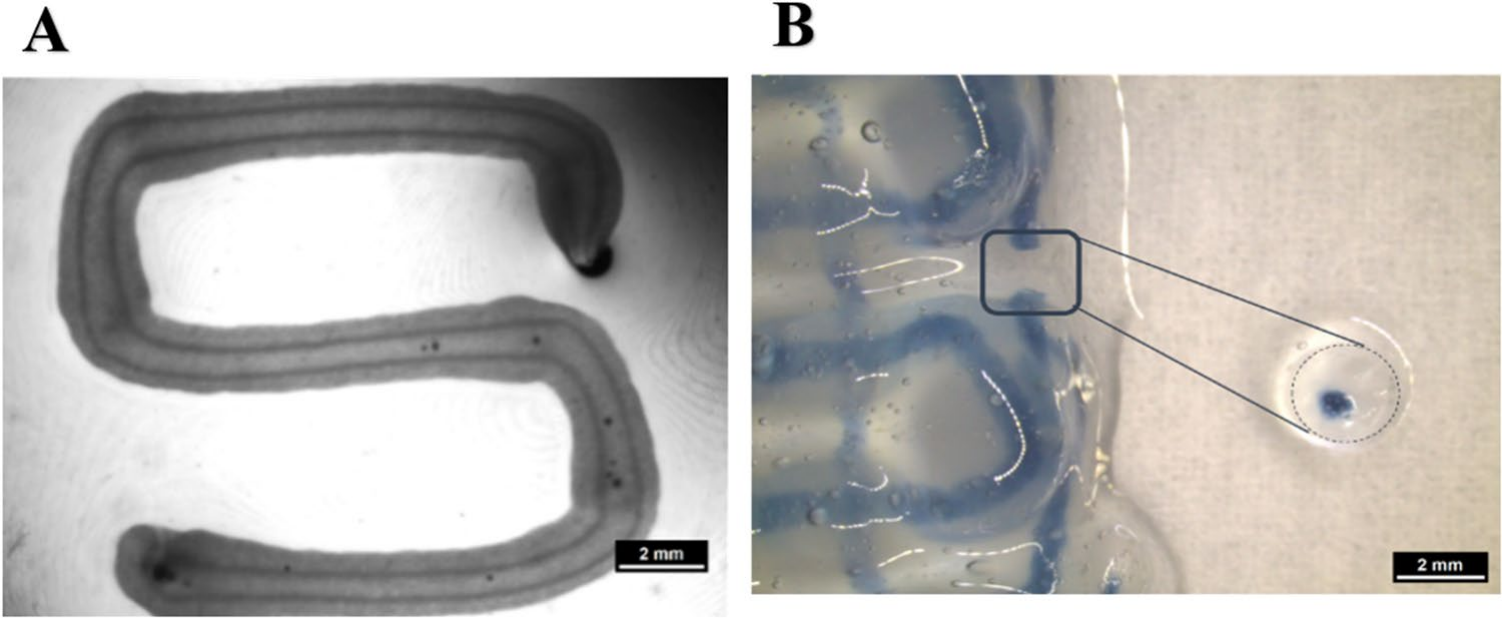
Core–shell plotting using 800 µm needle for shell and 400 µm needle for core. (**A**) Stereo microscopic image of a cell-free core–shell strand, deposited in meandering shape, with clear separation of core (algMC) and shell (algMC + Matrigel) compartments within the strand; scale bar 2 mm. (**B**) Stereo microscopic image of a cell-free, printed scaffold with blue stain in the core for visualizing the continuous core compartment through the coaxial strand; The insert shows a section through the strand segment, cut out from the scaffold scale bar 2 mm.

Core–shell strands were fabricated using coaxial needles of 800 µm (shell) and 400 µm (core) outlet diameter. As shown in Fig. 7A, after plotting and crosslinking, the Matrigel-supplemented algMC ink in the shell compartment was clearly separated from the core compartment which comprised of algMC only. Scaffolds consisting of core–shell strands (Fig. 7B) were deposited in a layer-by-layer fashion with a pre-defined geometry and showed high stability and shape fidelity after printing and crosslinking which is maintained during cultivation and withstood the multi-step staining procedure during analysis.

**Figure 8:**
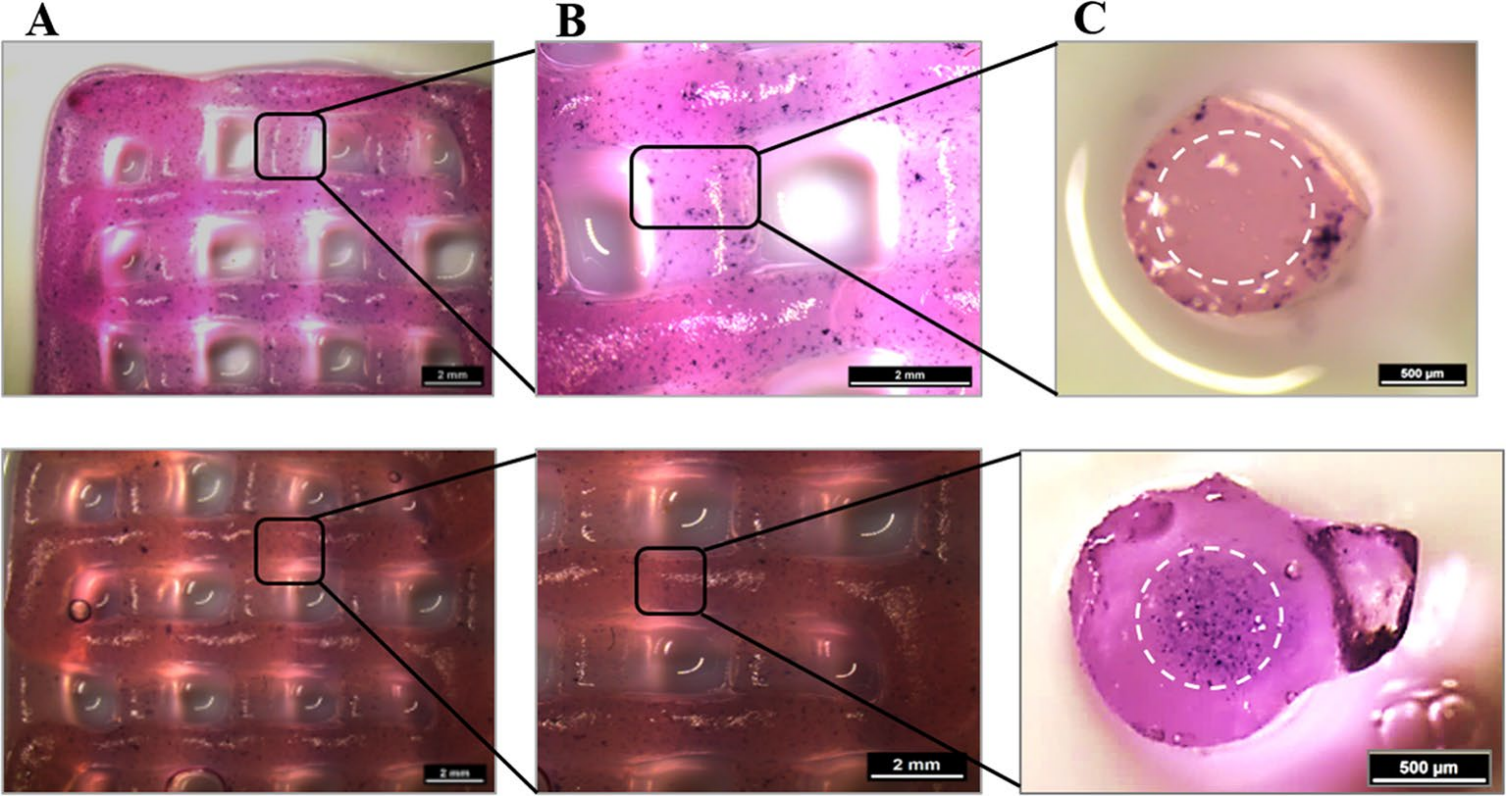
Metabolic activity at day 7 of cultivation (via MTT stain; violet) of HepG2 and NIH 3T3 cells embedded in core–shell printed hydrogel scaffolds; the core–shell interface is visualized via white dashed lines in **C**. Upper tile shows HepG2 encapsulated in the shell compartment consisting of algMC + Matrigel whereas the core remained cell free. Lower tile shows NIH 3T3 cells encapsulated in the core compartment consisting of algMC whereas the shell remained cell-free. (**A**,**B**) Scale bars 2 mm. (**C**) Scale bars 500 µm.

In order to investigate the influence of extrusion through coaxial needles on localization of encapsulated cells, HepG2-laden algMC + Matrigel was printed as shell bioink in combination with a cell-free algMC core material (Fig. 8A) and NIH 3T3-laden algMC was printed as core bioink with cell-free algMC + Matrigel as shell material (Fig. 8B). Scaffolds printed and crosslinked were mechanically robust also in presence of the cells while the strands maintained their cylindrical shape, defined coaxial structure and open macropores of the scaffolds over the whole culture period of 14 days. MTT assay, performed after 7 days of culture, indicated metabolically active cells in their respective compartments, stained by a violet color (Fig. 8).

#### Simultaneous embedding of HepG2 and NIH 3T3 cells in core–shell strand scaffolds

In order to visualize both cell types after simultaneous bioprinting, first co-culture experiments were conducted with pre-labeled cells: DiD-labeled hepatocytes (red) were encapsulated in shell compartment consisting of algMC + Matrigel while DiI-labeled fibroblasts (cyan) were encapsulated in the core consisting of algMC. These scaffolds were then compared to monoculture scaffolds which had DiD-labeled hepatocytes in shell only with no cells in the core.

**Figure 9:**
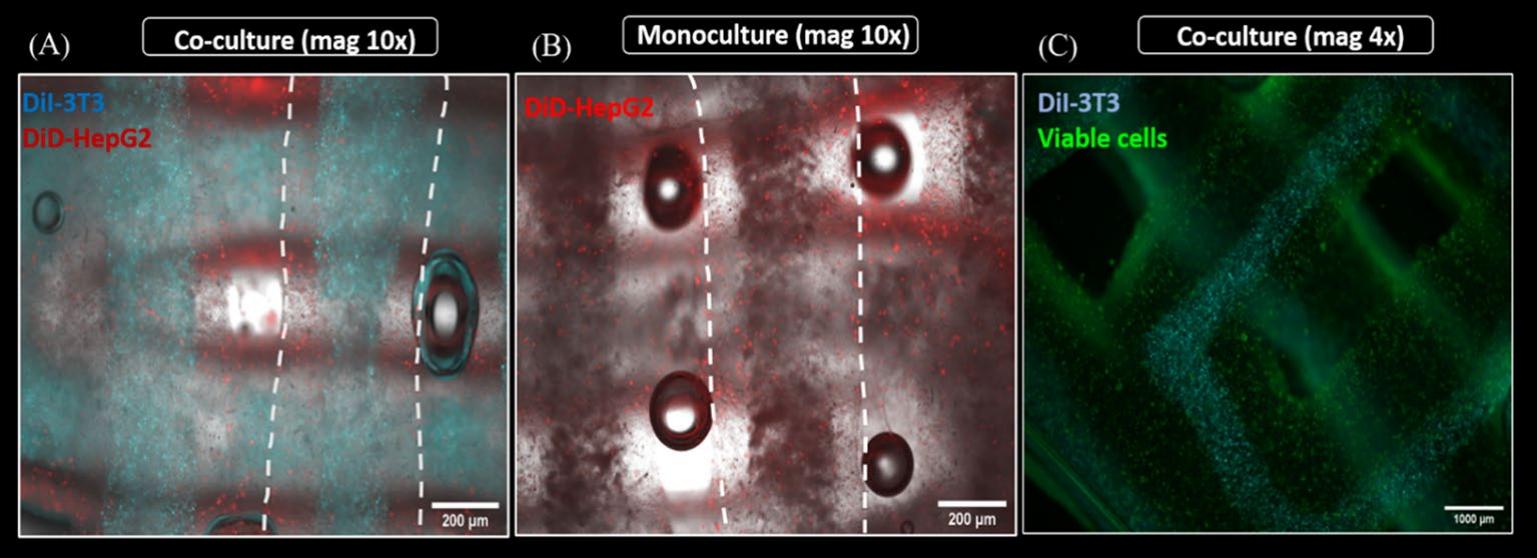
Spatially defined distribution of HepG2 and NIH 3T3 in core–shell strand scaffolds. (**A**,**B**) Fluorescence images of strands in a scaffold illustrating distribution of encapsulated cells in HepG2/NIH 3T3 co-culture vs. HepG2 monoculture scaffolds; scale bar 200 µm. White dashed lines indicate the whole strand width. (**C**) Fluorescence image of core–shell strand co-culture scaffolds showing Calcein-stained viable cells in green on day 4 of cultivation; for visualization of the core, the cyan channel (DiI-labeled NIH 3T3) was overlaid; scale bar 1000 µm.

Figure 9 clearly illustrates the distribution of cells along the whole strand in HepG2/NIH 3T3 co-culture (Fig. 9A) vs. HepG2 monoculture (Fig. 9B). By staining the core–shell strand scaffolds with Calcein AM, the viability of both cell types in the co-culture was proven—for better visualization, channels of calcein (green) and DiI (cyan, indicating the fibroblasts in the core) were merged in Fig. 9C.

**Figure 10:**
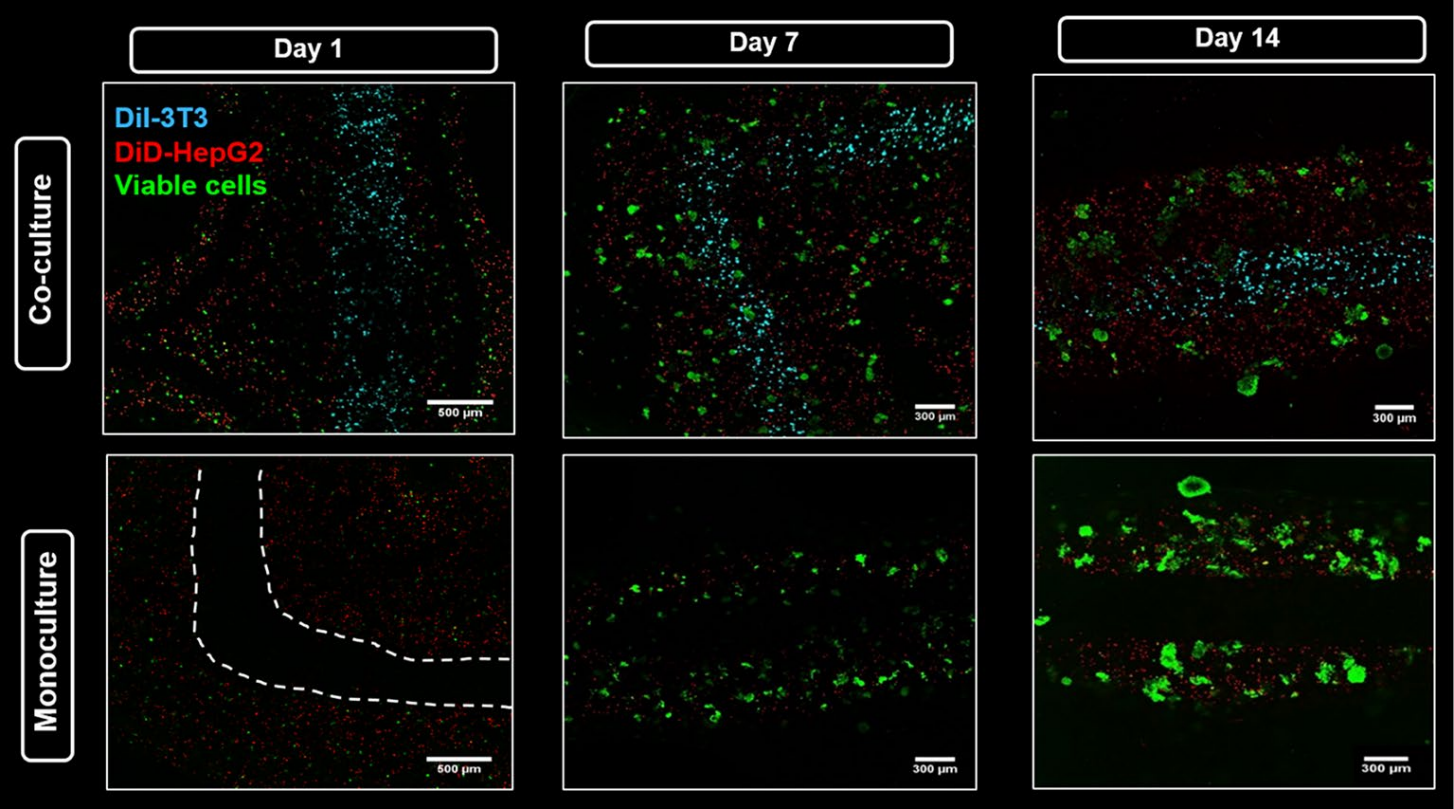
Viability and localization of HepG2 in co-culture with NIH 3T3 and in monoculture in core–shell strand scaffolds. Confocal images show DiD-labeled HepG2 (red) encapsulated in shell compartment consisting of algMC + Matrigel with either NIH 3T3 fibroblasts encapsulated in algMC core and labeled with DiI (cyan) (upper tile) or with a cell-free algMC core (lower tile). Viable cells were stained with Calcein-AM (green). Figure shows the progression of cell cluster formation of hepatocytes over the culture period. Scale bar (day 1) 500 µm; scale bar (days 7 and 14) 300 µm; white dashed line indicates here the core–shell interface.

Confocal fluorescence images acquired for both, co-culture and monoculture core–shell strand scaffolds, showed viable cells in both conditions forming clusters from single cells at day 1 as they were cultured until day 14. Thus, proliferation of HepG2 as well as maintenance of their viability and localization in the shell compartment was indicated (Fig. 10). On the other hand, NIH 3T3 fibroblasts encapsulated in algMC core had a round shape throughout the culture period, suggesting that pure algMC does not support the typical phenotype of fibroblasts which grow in an elongated spindle-shape morphology forming cell–cell connections. Besides this, maintenance of their localization in the core compartment was observed, too (Fig. 10).

#### Functionalization of core bioink – enhancing fibroblast network formation

**Figure 11:**
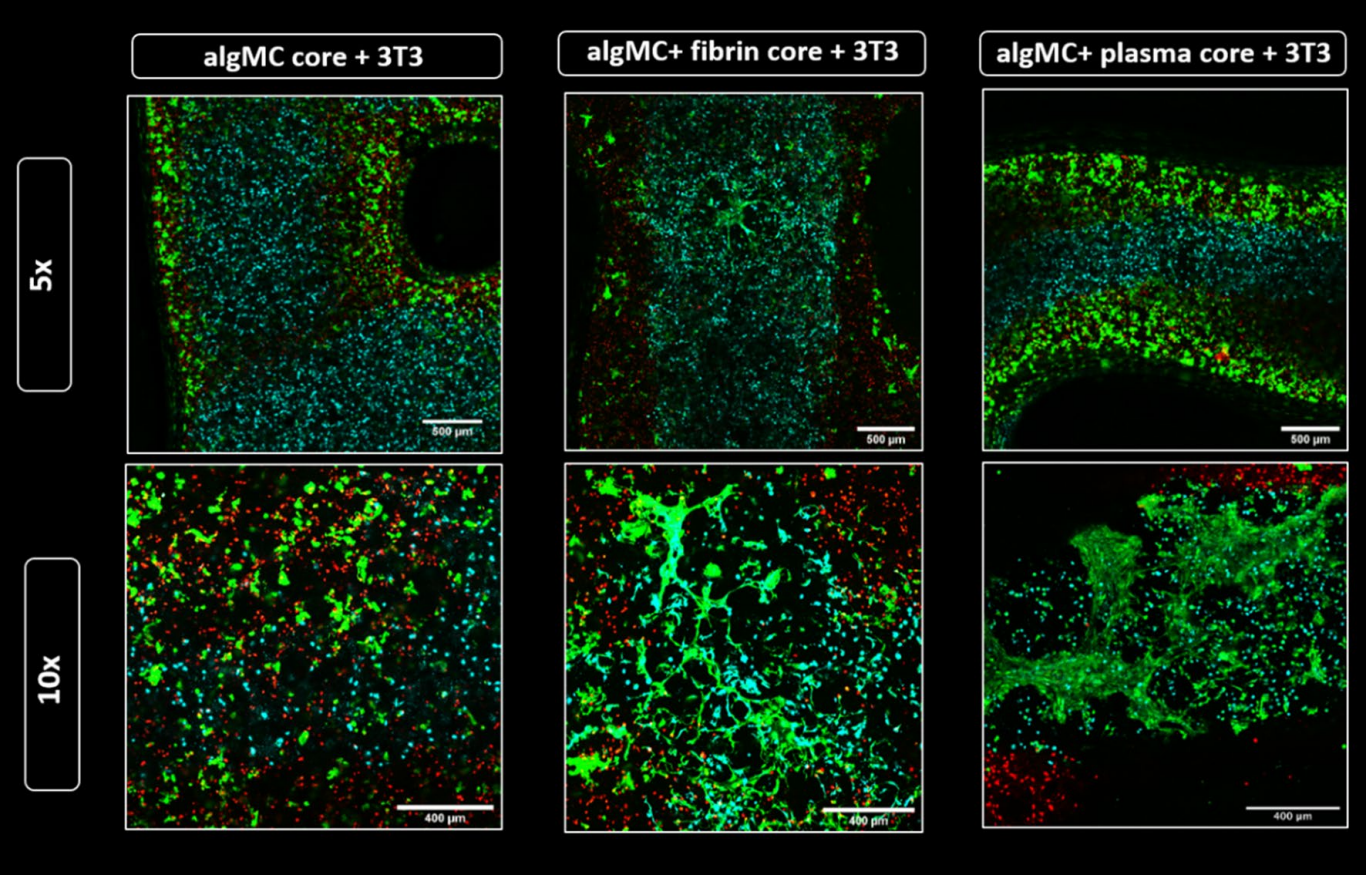
Fibroblasts network formation in the core compartment at day 7 of co-culture. Confocal images of core–shell hydrogel strands with encapsulated DiD-labeled HepG2 (red) in algMC + Matrigel shell and DiI-labeled NIH 3T3 fibroblasts (cyan) embedded in the core of algMC, algMC supplemented with fibrin and algMC supplemented with plasma. Viable cells are stained green with Calcein AM, indicating formation of fibroblast networks within the fibrin- and plasma-functionalized core while a round morphology of the fibroblasts is maintained in unmodified algMC core. Upper tile gives an overview of a section of the core–shell strand; magnification 5x, scale bar 500 µm. Lower tile shows higher magnification (10x) images of NIH 3T3 fibroblasts in the core; Scale bars 400 µm.

As we hypothesized that the phenotype of the fibroblasts has a direct impact on the interaction with the hepatocytes, the next step was investigating the effect of functionalized bioinks on NIH 3T3 phenotype. Therefore, algMC as base core ink was supplemented with either fibrin or human blood plasma to provide ECM components with cell and protein binding sites as well as, in case of plasma, growth factors. With this functionalization, we intended to enhance NIH 3T3 cell adhesion to matrix, proliferation and cell–cell interaction resulting in network forming cell phenotype in the core. Results in Fig. 11 clearly show spreading and attachment of the fibroblasts after 7 days of cultivation within algMC + fibrin and algMC + plasma: the fibroblasts formed extended networks between each other within the core while we observed even direct interactions at the interface between core and shell with the hepatocytes in some areas along the strand. In contrast, fibroblasts in the non-functionalized algMC core remained in a round morphology, showing no sign of network formation.

### Influence of the microenvironment on expression of hepatic marker proteins in the core–shell bioprinted co-culture system

Hepatocytes functionality is often assessed by their ability to synthesize and secrete specific proteins. Therefore, in order to investigate the effect of the fibroblasts grown in different core materials on the hepatocytes in the neighboring shell compartment, the expression of specific marker proteins in the different conditions was observed by antibody staining and subsequent immunofluorescence microscopy. The first marker which was analyzed, albumin (Alb), is exclusively synthesized by hepatocytes. With cytokeratin-19 (CK-19), a hepatobiliary/progenitor cell marker has been assessed which is also expressed in HepG2 in response to specific signaling factors^34,35^. Finally, α-1 antitrypsin (AAT) was analyzed, which is a protease marker also released in inflammatory reactions and a biomarker for liver injury diagnosis. Cytoskeletons and nuclei were stained in combination with each marker in order to visualize functional clusters. Printed and crosslinked core–shell scaffolds were analyzed after 14 days of cultivation. In all conditions, HepG2 were embedded in the shell consisting of algMC + Matrigel; for the core, the following conditions were compared: (I) algMC w/o NIH 3T3 (HepG2 monoculture as control), (II) algMC with NIH 3T3, (III) algMC + fibrin with NIH 3T3 and (IV) algMC + plasma with NIH 3T3.

**Figure 12:**
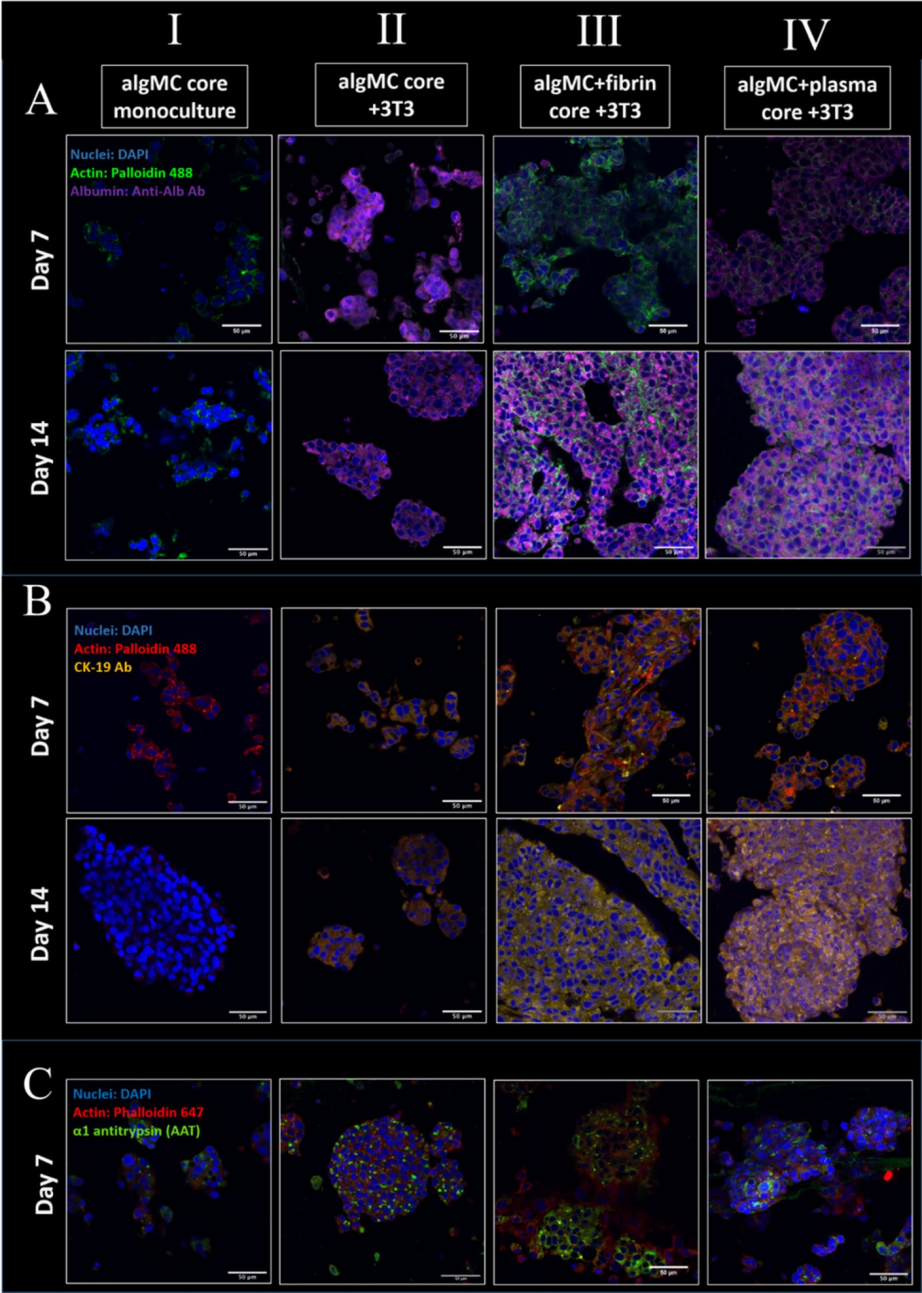
Cluster formation and biomarker expression of HepG2 embedded in shell compartment in monoculture (cell-free algMC core; I) and in co-culture with NIH 3T3 fibroblasts embedded in core compartment of different compositions (II-IV). (**A**) Confocal images of HepG2 stained for Albumin (purple), nuclei (blue) and cytoskeletons (green); scale bars 50 µm. (**B**) Confocal images of HepG2 stained for CK-19 (yellow), nuclei (blue) and cytoskeletons (red); scale bars 50 µm. (**C**) Confocal images of HepG2 stained for AAT (green), nuclei (blue) and cytoskeletons (red); scale bars 50 µm.

From the assessment of each biomarker (Fig. 12A–C), it could be observed that the hepatocytes proliferated in all conditions to form multicellular clusters. The size and interconnection of the clusters increased over time (shown in Fig. 12: between day 7 and day 14 of cultivation). Moreover, the cluster size—and therefore proliferation—was enhanced in co-culture with NIH 3T3, especially after enriching the core bioink with fibrin or plasma: in these cases, fusion of the aggregates resulting in even bigger clusters (Fig. 12). The intensity of the biomarker stainings differed considerably between the conditions. Albumin showed a low expression level in monoculture scaffolds (Fig. 12A I) (for visualization of separate channels, refer to supplementary information Fig. S3), whereas high expression levels were observed in all co-culture conditions (Fig. 12A II–IV). The expression level of CK-19 was very low in the case of monoculture (Fig. 12B I; for visualization of separate channels, refer to supplementary information Fig. S4) unlike for all co-culture conditions. This increase of expression level was even more pronounced for fibrin and plasma supported core bioinks (Fig. 12B II–IV). Finally, the AAT biomarker showed expression in all conditions but was higher in presence of fibrin in the core (Fig. 12C III).

**Figure 13:**
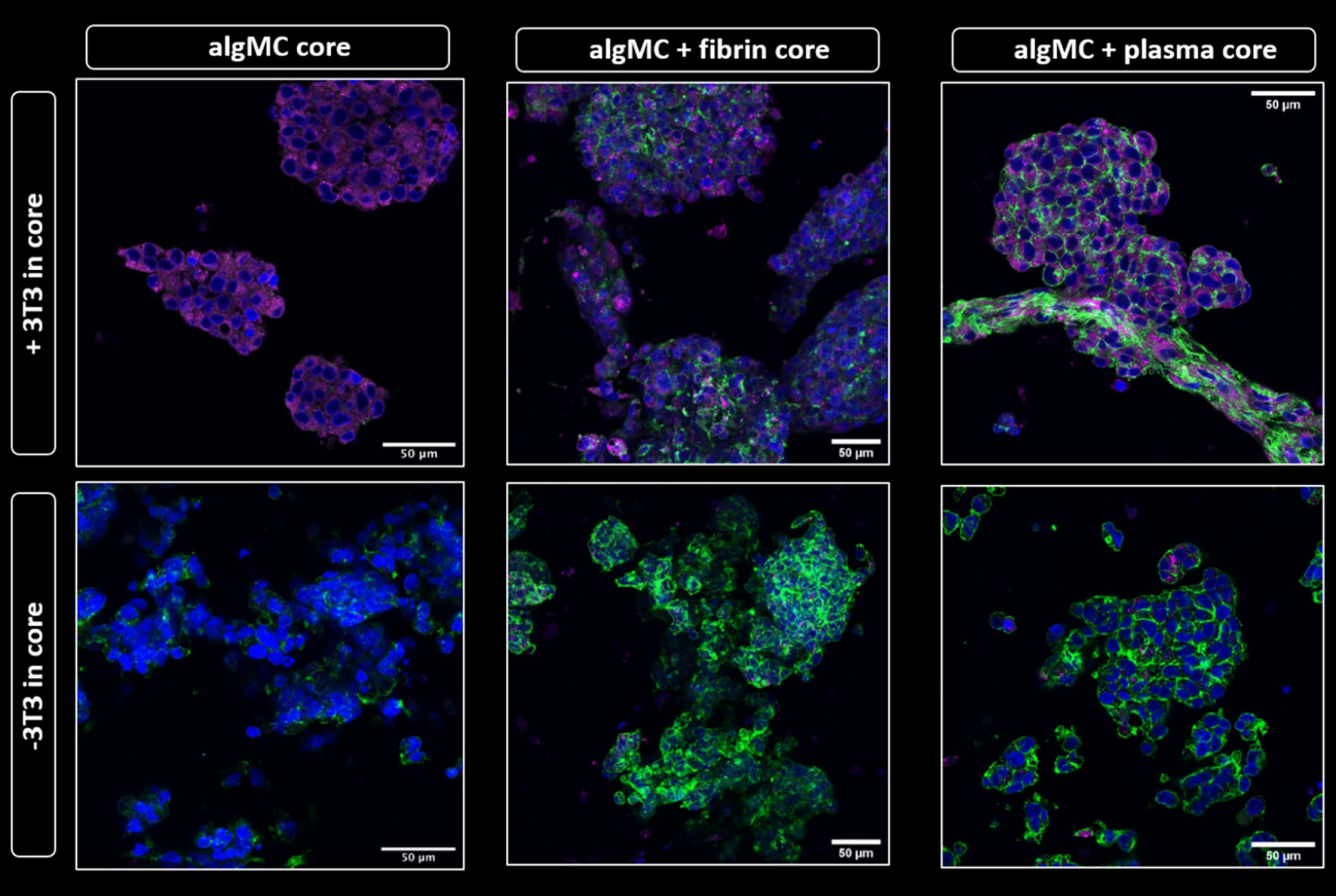
Albumin expression of HepG2 clusters formed in shell compartment in co-culture with NIH 3T3 embedded in core compartment (upper panel) and in monoculture (cell-free core; lower panel). In both, co-culture and monoculture, the core was composed of algMC, algMC + fibrin and algMC + plasma. Confocal images of HepG2 stained for Albumin (purple), nuclei (blue) and cytoskeletons (green); scale bars represent 50 µm.

The data shown in Fig. 12 indicated a strong influence of the fibroblasts embedded in the fibrin and plasma supplemented cores on the phenotype of the hepatocytes embedded in the shell. It was therefore important to investigate whether this effect was due to the core material itself and the factors that it offers or due to the enhanced fibroblasts performance in these bioinks with regards to their spreading, proliferation and matrix formation. To answer this question, an experiment was conducted comparing the three different core materials—algMC, algMC + fibrin and algMC + plasma—in presence and absence of NIH 3T3 fibroblasts in the core. The effect of these mono- and co-culture conditions on hepatocyte cluster formation and albumin expression was studied. Fluorescence images revealed that the expression of albumin was strongly enhanced in the co-culture with NIH 3T3 in comparison to monocultures, shown by the higher intensity of albumin expression with all different core compositions (Fig. 13). This observation, that the presence of the fibroblasts in the core is essential for the increased biomarker expression, was also proven for CK19 (supplementary data Fig. S6). Another interesting observation was the cell–cell interactions at some sites of the interface between core and shell compartments within one strand, as exemplarily shown in Fig. 13 in the upper right panel for the interconnection between the hepatocytes forming clusters in the shell and the fiber-like networks of fibroblasts in the core.

### Rheological and mechanical properties of the developed hydrogel system

#### Rheological characterization of the modified inks

**Figure 14:**
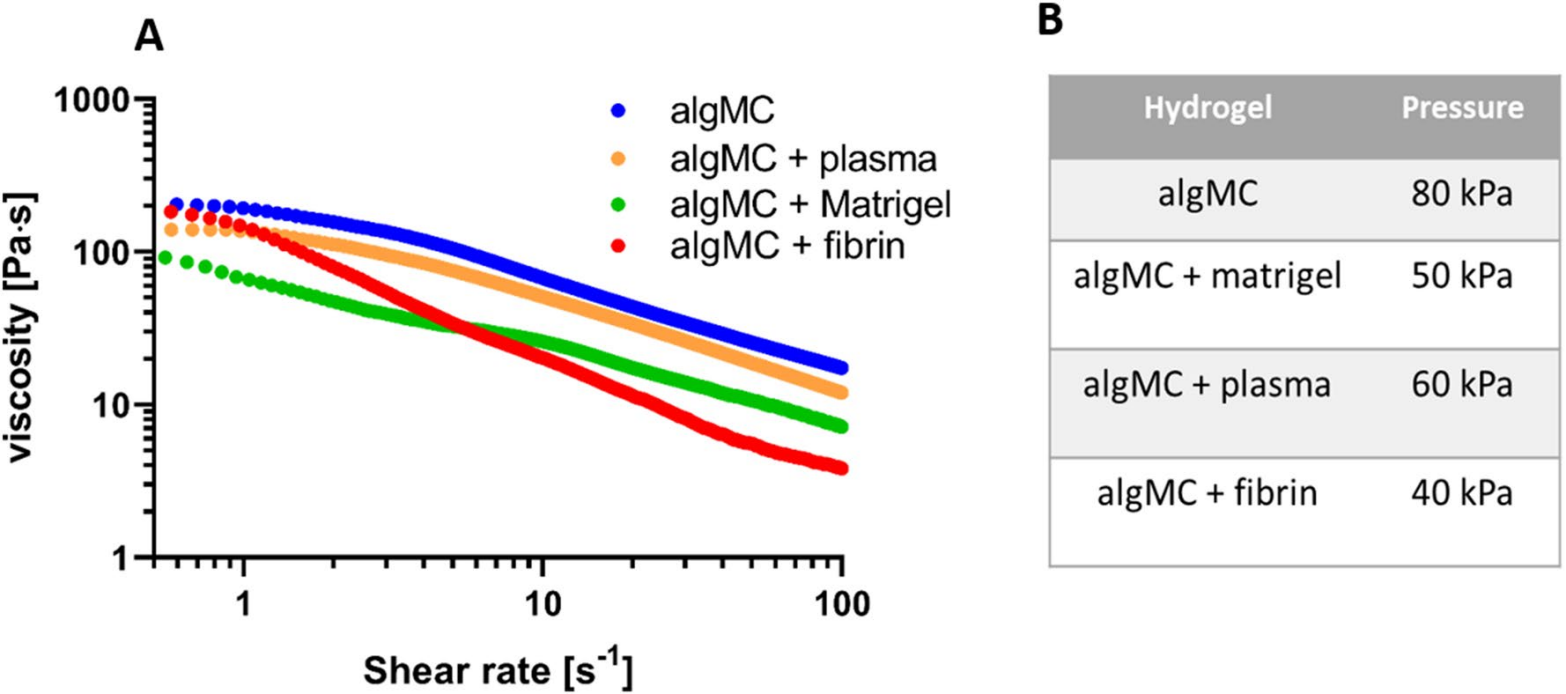
Rheological characterization of functionalized algMC and printing pressure adaptation. (**A**) Representative curves of shear thinning behavior of algMC-based inks at room temperature. (**B**) Printing pressure used for the different bioinks.

Rheological analysis of all four hydrogel blends used in this study demonstrated shear thinning behavior at increasing shear rates (1–100 s^−1^, Fig. 14A), enabling extrusion through a printing nozzle and shape retention after printing. The graph illustrates that unmodified algMC shows the highest viscosity while the viscosity was decreased after addition of cell-supportive supplements to the algMC bioink (matrigel, plasma, fibrin). Accordingly, the air pressure applied for extrusion of accurate and stable strands of modified algMC inks was distinctly lower than for unmodified algMC (Fig. 14B).

#### Mechanical characterization of monophasic and core–shell strand scaffolds printed from the modified inks

**Figure 15:**
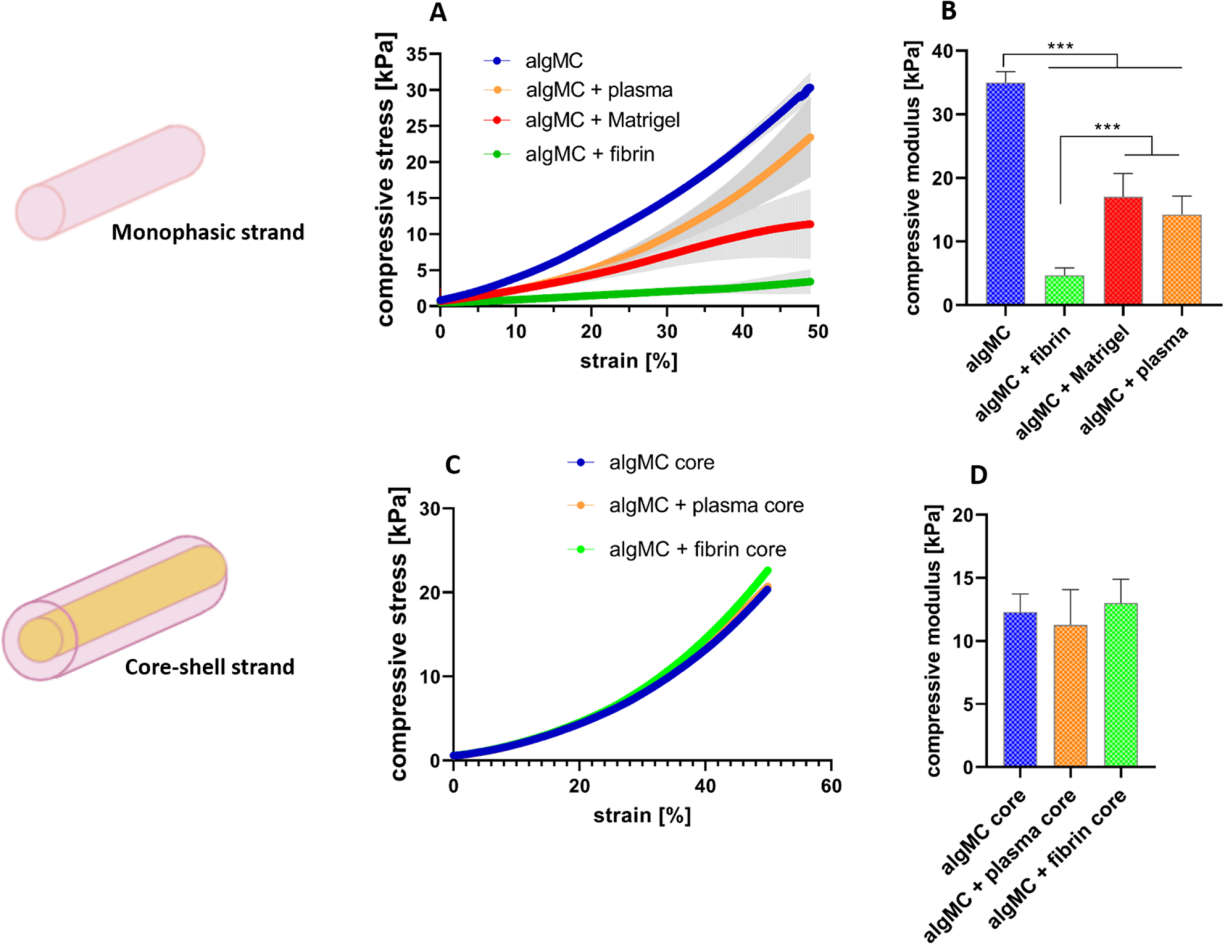
Mechanical characterization of monophasic and core–shell strand scaffolds after printing and crosslinking. (**A**) Stress–strain curves of cross-linked monophasic scaffolds at day1. (**B**) Compressive modulus of cross-linked monophasic scaffolds at day 1. (**C**) Stress–strain curves of cross-linked core–shell scaffolds with varying core compositions in algMC + Matrigel shell at day1. (**D**) Compressive modulus of cross-linked core–shell scaffolds at day 1 (n = 4, p*** < 0.0005, mean ± SD).

In order to further characterize the modified hydrogel blends regarding their post-printing and -crosslinking properties, uniaxial compressive tests were performed for monophasic scaffolds to study the mechanical stability and stiffness of 3D constructs after plotting and post-processing (Fig. 15A,B). Based on compressive stress–strain curves for monophasic printed scaffolds, the compressive modulus was determined to be highest for plain algMC compared to the modified algMC blends indicating that it possesses the highest stiffness of the four hydrogels. Fibrin-supplemented algMC specifically revealed the lowest compressive modulus indicating that the 3D network possesses the lowest stiffness. Matrigel- and plasma-functionalized algMC blends showed comparable compressive moduli ranging in between algMC and algMC + fibrin.

Measuring the mechanical properties of core–shell strand scaffolds with algMC + Matrigel as shell material and either algMC, algMC + plasma, or algMC + fibrin as core revealed no significant differences between the different scaffold types in the stress–strain curves and calculated compressive moduli, suggesting that the mechanical features of scaffolds are mainly owed to the shell biomaterial (Fig. 15C,D).

## Discussion

For the development of specific tissue models that can be used as in vitro disease models or for pharmacological testing studies, extrusion-based 3D bioprinting is a powerful tool in the generation of relevant tissue-like constructs. Towards development of a 3D liver model, the main goal of our work was mimicking the physiological microenvironment in suitable bioinks for encapsulated cells in different compartments, to enable the generation of functional 3D tissue constructs.

In the current study, the first aim was developing and evaluating a hydrogel based system that supports bioprinting of hepatocytes and maintains their viability and function in its specific 3D environment in vitro. HepG2 is a well-established cell line often used for in vitro modelling for human hepatocytes^36,37^. To this end, algMC, a cytocompatible ink with excellent printing properties^17^, was supplemented with Matrigel which is rich in complex extracellular matrix proteins and growth factors. Matrigel concentration in the ink was optimized to be 5%; a lower concentration did not show the desired positive impact on the cells and higher concentrations of 10% and 20% did not provide scaffolds which were stable throughout the cultivation time (data not shown). The addition of Matrigel resulted in an enhanced viability, spreading and proliferation of the hepatocytes (Figs. 1, 2 and 4) which might be mediated and triggered by the ECM proteins and growth factors provided by Matrigel and/or via altering the alginate network strength and structure. Results also indicate that the hepatocytes developed over time from single cells to converged cell clusters and further to multicellular spheroids which remained viable and stable throughout the study while being homogeneously distributed within the 3D scaffold. The cluster formation was clearly supported by Matrigel as bigger clusters with a more pronounced actin structure were detected in comparison to Matrigel-free algMC (Figs. 1 and 4). HepG2 were found to maintain an active, quantifiable albumin production during the cultivation time that was, however, not improved in Matrigel-functionalized algMC (Fig. 5). Matrigel was utilized in previous studies using hepatocytes to show its cell supportive function in non-bioprinted 3D systems^38^ while one team described how Matrigel cultured hepatocytes formed a continuous network of functional proto-bile canaliculi structures in plain Matrigel structures for in vitro virus infectivity tests^39^. Previous studies showed that 3D bioprinting of hepatocytes can be a valuable and potent tool to recapitulate the cells’ in vitro microenvironment^11,40,41^; Hiller et al.^40^, Zhu et al.^41^ and Mistry et al.^11^ confirmed the suitability of alginate as base for a hepatocyte-comprising bioink.

As our approach was focused towards the generation of versatile, volumetric and fully functional co-culture models^42^, the second part of the study was to develop a structurally defined co-culture system to provide a more complex microenvironment for the hepatocytes by employing core–shell bioprinting, a useful tool which allows coaxial printing of two bioinks. Core–shell design ensures high contact areas between the different phases. Therefore, two cell types are brought in close proximity, thus allowing their interaction, and provides various options to mimic spatial arrangement of different cell types in a specific tissue microarchitecture. The conventional idea of coaxial bioprinting was to design strands that are made in a way to use a stiffer biomaterial ink for the shell to provide sufficient mechanical support of the core material which then can typically be a bioink of low viscosity to improve conditions for encapsulated cells^11,43^. For example, Mistry and colleagues demonstrated the cell encapsulation in core and shell compartments of coaxially extruded strands, however, in their approach the core ink consisted of low viscosity biomaterials (e.g. pure Matrigel) so that only the shell material contribute to mechanical stability of the scaffolds^11^. Other setups include printing a mechano-supportive core for cell-laden shell^44^ or even encapsulating cells in one compartment and dissolving the other compartment after cells have integrated together in the shell forming an initial tissue-like structure^45,46^.

In our study, we applied a concept of two independently stable yet cell supportive bioinks for core and shell, enabling us to flexibly combine different cell types in core and shell independent of fabrication considerations. No additional stabilization for the scaffolds, strand and distinct core–shell compartments should be required. Therefore, we aimed to create a modular and tailorable bioink system: We used algMC as a base material, fulfilling the essential requirements of printability and stability of overall scaffolds and accuracy of coaxial strand architecture, for both compartments and adjusted the ink properties with respect to cell-supportive effects. Thus, biological components were added for individual functionalization of algMC in the core and shell compartments, respectively, with the aim of improving cell attachment and function while maintaining printability and shape fidelity of the scaffolds. Rheological analysis revealed that the addition of cell-supportive supplements (matrigel, plasma, fibrin) to algMC led to a lower viscosity of the inks compared to plain algMC, however, print fidelity is maintained by adjusting the printing pressure (Fig. 14). After printing and crosslinking, these modifications of the algMC bioink resulted in haptically softer yet stable hydrogels that was confirmed by mechanical measurement: there were significant differences in the compressive moduli between plain algMC and the supplemented algMC bioinks (Fig. 15B). The lowest stiffness of algMC + fibrin scaffolds might be owed to the altered network density after preparation of the gel that includes mixing of fibrinogen and alginate in a 1:1-ratio. However, even this bioink produces stable scaffolds without the need of further stabilizing components. The softer hydrogels could contribute to a more favorable microenvironment for the hepatocytes since it was shown in a previous study that softer materials with high porosity proved to be more favorable for HepG2 cell proliferation^47^. When core–shell scaffolds with different inks as core materials were mechanically tested, no significant differences were observed (Fig. 15C,D), suggesting that mainly the shell material (algMC + Matrigel) contributes to the mechanical properties of the core–shell scaffolds. It can be concluded from these results that a modular system with independently stable and printable bioinks was developed with maintaining optimal functionality for the encapsulated cells.

In order to develop core–shell bioprinting towards the generation of functional liver models, we focused on the establishment of a spatially defined co-culture of hepatocytes with fibroblasts, based on previously described work: Lee et al. investigated a co-culture of HepG2 spheroids in indirect contact with NIH 3T3 fibroblasts in a micropatterned, fibrous scaffold separating the two cell lines and found a positive influence of the interaction of both cell types on hepatocytes function regarding albumin secretion. They hypothesized that this effect is owed to the diffusion of soluble factors or signaling molecules produced by the fibroblasts through the medium^30^. He and co-workers. observed an enhanced survival and albumin secretion of HepG2 in a direct contact co-culture with NIH 3T3 fibroblasts in origami-based 3D microstructures using alginate as a sacrificial biomaterial^32^. In addition, Mittal et al. investigated the effect of co-culturing primary human hepatocytes as spheroids (either mixed or layered) with NIH 3T3 fibroblasts and observed a 3- to fivefold increase in CYP (cytochrome p450) activity of the hepatocytes in comparison to monoculture conditions^33^.

In the present study, NIH 3T3 cells were encapsulated in algMC which constituted the core while HepG2 were encapsulated in algMC + Matrigel constituting the shell of the strand being in a coaxial co-culture with an interface between core and shell. By applying core–shell bioprinting through coaxial needles, we could show that both cell types survived the printing process and maintained their viability in the core and the shell compartment as well as the localization in their respective compartments during the subsequent cultivation, suggesting initial success of the core–shell system in establishing indirect co-culture conditions (Figs. 7, 8). The coaxial printing process did not cause a higher rate of cell death, as demonstrated by analysing the viability of core–shell vs. monophasic (full-strand) algMC scaffolds via live/dead staining at day 1 after printing for HepG2 cells in shell (Supplementary Fig. S7). The recovery of the cells from the shear stress during extrusion appeared to be similar in both conditions although it is expected that in core–shell needles cells experience more shear stress due to the design of the needles: As recently shown by our group in a simulation study based on numerical and analytical modelling, the shear stress is highest close to the nozzle walls^48^—in case of the coaxial extrusion, the contact area of the shell bioink to the nozzle wall is higher compared to monoaxial extrusion. On the other hand, cells can resist considerably high shear stress if they are exposed to it only for a short time^49,50^.

Since alginate lacks specific binding sites for mammalian cellular attachment, fibroblasts cannot develop their normal spindle-shaped, elongated morphology or attach to the surrounding matrix; in the plain algMC bioink as a core, they appeared rather roundish in shape throughout the culture period. As we expected that the positive effect of fibroblasts on hepatocytes depends on a physiological morphology, the third part of the study was focused on optimizing the core biomaterial to support the attachment and spread of encapsulated fibroblasts. This was achieved by functionalizing the algMC blend of the core with either fibrin or human plasma. The used plasma contains about 2.26 mg ml^−1^ fibrinogen and other structural proteins as well as signaling factors which has been shown to enhance spreading and proliferation of primary osteoprogenitor cells as shown recently by Ahlfeld et al.^21^. Herein, we were able to demonstrate that the addition of these components to the basic algMC ink could successfully allow the attachment and spread of fibroblasts which exhibited their fiber-like morphology and formed interconnecting fibrous networks extending to the neighboring shell compartment (Fig. 11).

In the fourth part of the study, the formation of functional clusters of HepG2 in dependence of the microenvironment was analyzed by applying the established core–shell bioprinting based co-culture system. Results of the immunofluorescence analyses shown in Figs. 12 and 13 revealed a considerable effect of the culture conditions on the hepatocytes. The presence of fibroblasts, even in round morphology in the algMC core, resulted in strongly increased expression levels of albumin and CK-19 as well as in a slight increase of the cluster size. The fibroblasts grown in fibrin or plasma functionalized algMC were observed to form interconnecting networks which induced a strong proliferation and finally formation of larger hepatocyte clusters (Fig. 12). In immunofluorescence images of hepatocytes clusters in plasma supplemented core (Fig. 13), in few locations we observed a direct interconnection between hepatocytes clusters and elongated structures of fibroblasts taking place at the interface of core and shell compartments. These results suggest the presence of interaction and cross-talk between the two cell types through communication via soluble factors. Those might diffuse through the algMC network while the two cell types remained in their respective compartments. Another reason might be the active fibroblasts interconnecting and forming matrix which can also influence the hepatocytes. Enhanced cluster formation of hepatocytes could be also due to the biochemical effects of ECM components provided from fibrin or plasma or produced in the core from fibroblasts.

**Figure 16:**
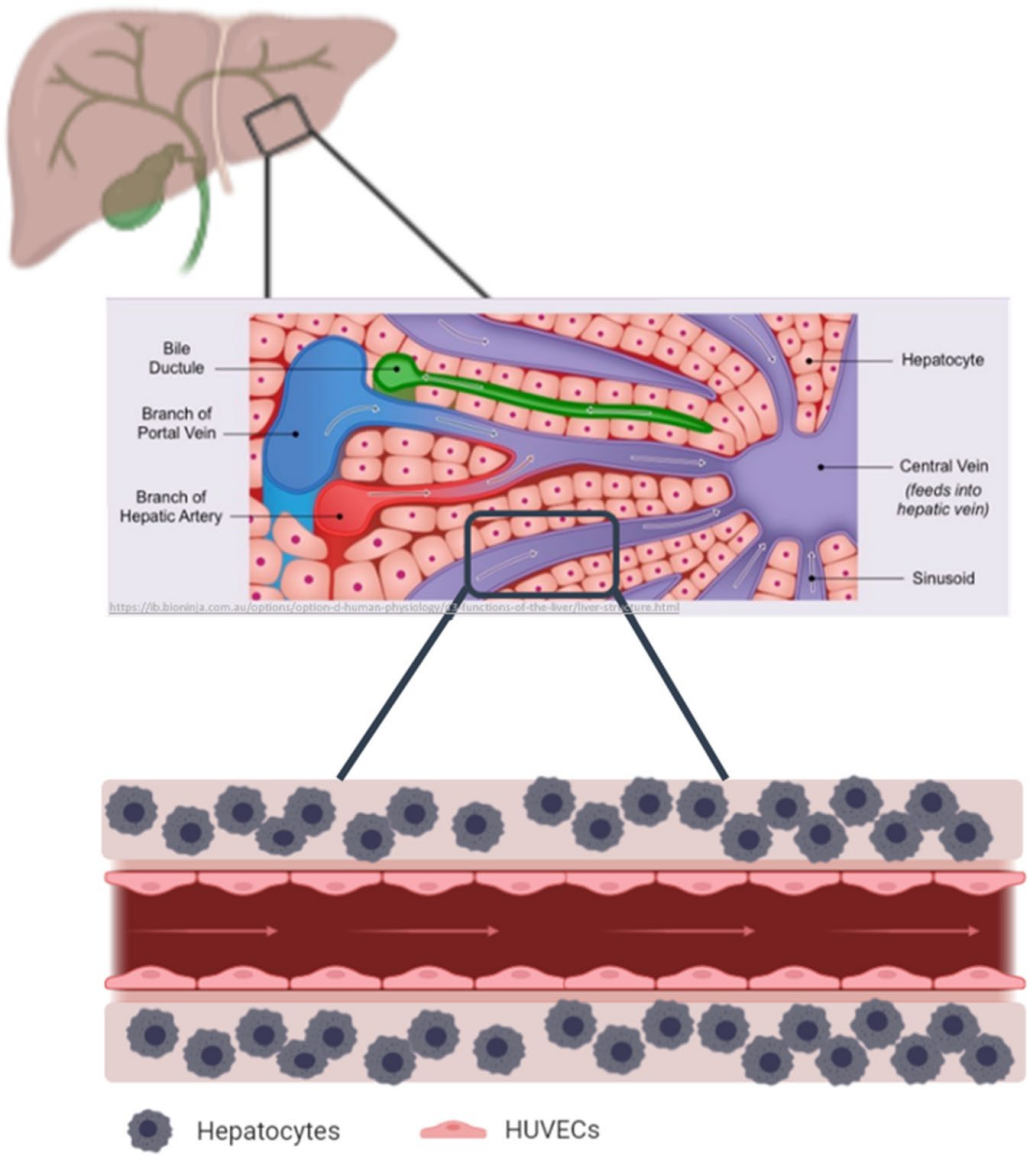
Graphical representation of the hepatocyte co-culture concept, showing how an in vivo liver lobule is consisting of blood vessels, ducts and canals (vasculature) that are surrounded by sheets of hepatic cells as shown in the upper illustration. Therefore, the concept of mimicking the microarchitecture of the liver lobule is envisaged to be represented in a coculture model of hepatocytes printed in the shell and endothelial cells lining the core of a perfusable construct (lower illustration). Middle illustration obtained from Cornell, B. 2016. Hepatic lobules. [ONLINE] Available at: http://ib.bioninja.com.au^51^.

With the aim of fabricating liver tissue models, co-culturing fibroblasts in core–shell fashion was the first step to provide insights into the great potential of this co-culture bioprinting strategy. Towards developing in vitro liver models, the development of co-culture systems with other cell types would be the aim of our future work after demonstrating here how using core–shell technology enabled us to engineer a tailored 3D microenvironment for the bioprinted cells. In order to mimic the physiological liver structures which constitutes of vasculature and interconnected vessels, the next steps would be to further increase the complexity developing printed constructs for co-cultivation of hepatocytes with endothelial cells and other supporting cell types. This will allow us to study the cellular interactions in these 3D systems and to establish perfusable liver models (Fig. 16).

## Conclusion

In this work, we have presented 3D extrusion-based bioprinting as a promising tool to generate biologically active liver tissue models. By specific adaptation of the bioink composition and by applying coaxial printing of two distinct bioinks in core–shell fashion, the 3D microenvironment of hepatocytes can be controlled and tuned to mimic specific conditions. With the presented method, we established a functional co-culture model of hepatocytes and fibroblasts with a core–shell strand/scaffold design. By comparing different ink compositions (algMC with and without bioactive components) and co-cultures vs. monocultures, we could demonstrate in our proof-of-concept study a strong influence of changing components of the spatially defined 3D microenvironment on hepatocyte proliferation and expression levels of selected hepatocyte-specific markers. These findings highlight the importance of tailoring the 3D microenvironment towards generation of artificial liver constructs and the high potential of versatile bioprinting technologies for that purpose.

## Supplementary Information

Supplementary Information.

## Data Availability

All experiments and methods were performed in accordance with relevant guidelines and regulations.
