# Supplementary material for: 3D bioprinting of hepatocytes: core–shell structured co-cultures with fibroblasts for enhanced functionality
Source: Sci Rep. 2021 Mar 4;11:5130. doi: 10.1038/s41598-021-84384-6 (PMC7933206; doi:10.1038/s41598-021-84384-6)
Supplement: Supplementary file 1 — Supplementary Information. [file 41598_2021_84384_MOESM1_ESM.pdf]

3D bioprinting of hepatocytes – core-shell structured co-cultures with fibroblasts for enhanced functionality

Rania Taymour, Michael Gelinsky, David Kilian, Tilman Ahlfeld, Anja Lode*

Centre for Translational Bone, Joint and Soft Tissue Research, Faculty of Medicine and University Hospital *Carl Gustav Carus*, TU Dresden, Germany

SUPPLEMENTARY INFORMATION

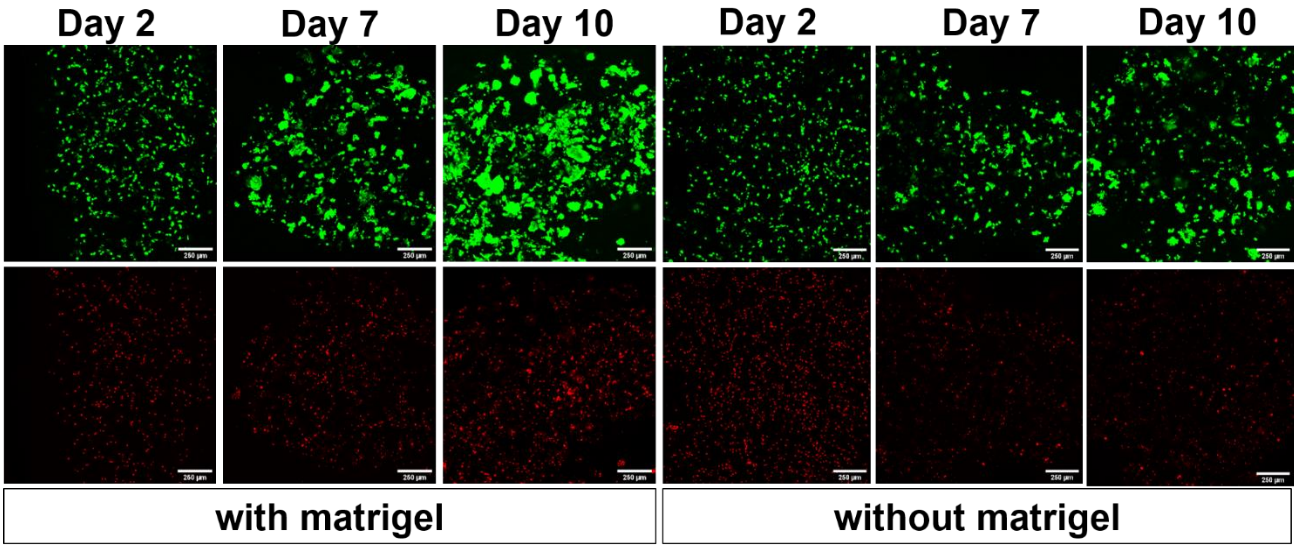

Supplementary figure S1: Viability of HepG2 cells embedded in algMC with and without Matrigel. cLSM fluorescence images of live/dead staining of HepG2 embedded in bioprinted scaffolds at day 2, 7, and 10 of cultivation. Viable cells stained green (Calcein, upper panel), whereas dead cells are stained red (ethidium homodimer-1, lower panel); scale bar = 250 µm.

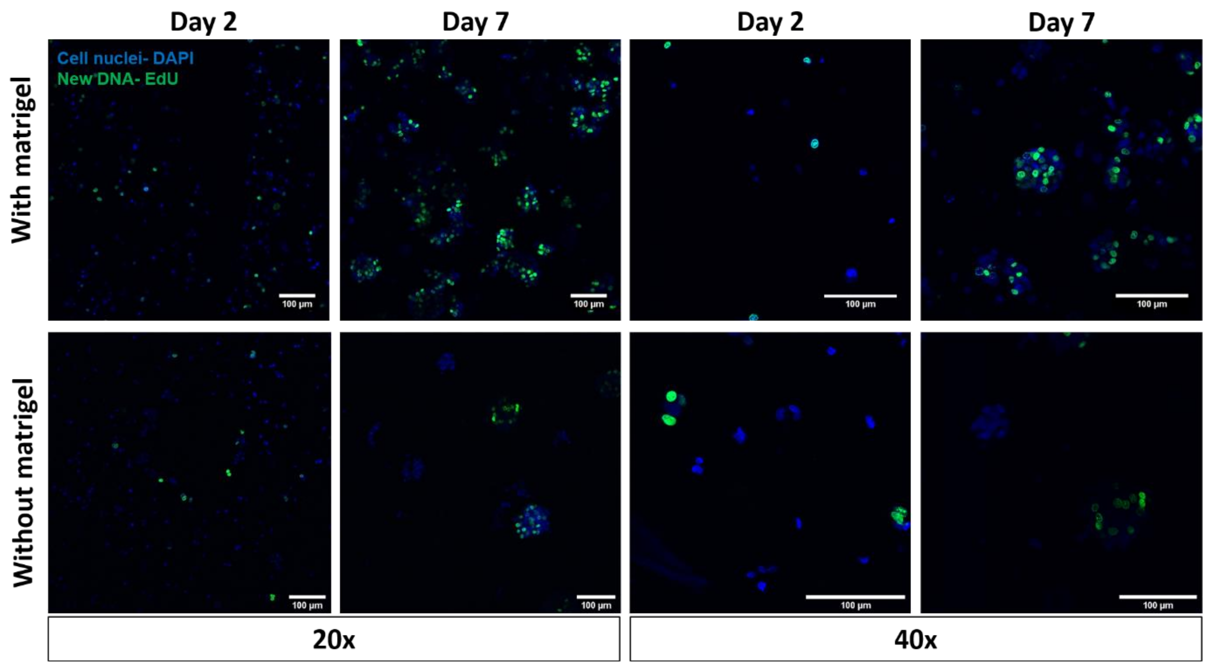

Supplementary figure S2. Proliferation of HepG2 embedded in algMC with Matrigel vs without matrigel – EdU proliferation assay. Cell nuclei were stained with DAPI (blue); representing original cells, while cells labelled with EdU are shown in green; representing newly formed DNA and therefore mitosis. Left tile (20x magnification) shows the distribution of newly formed cells allover a specific region of the scaffold in days 2, 7 of cultivation in scaffolds with and without matrigel. Right tile (40x magnification) shows distribution of cells within the formed clusters and their rate of proliferation; scale bars represent 100 µm.

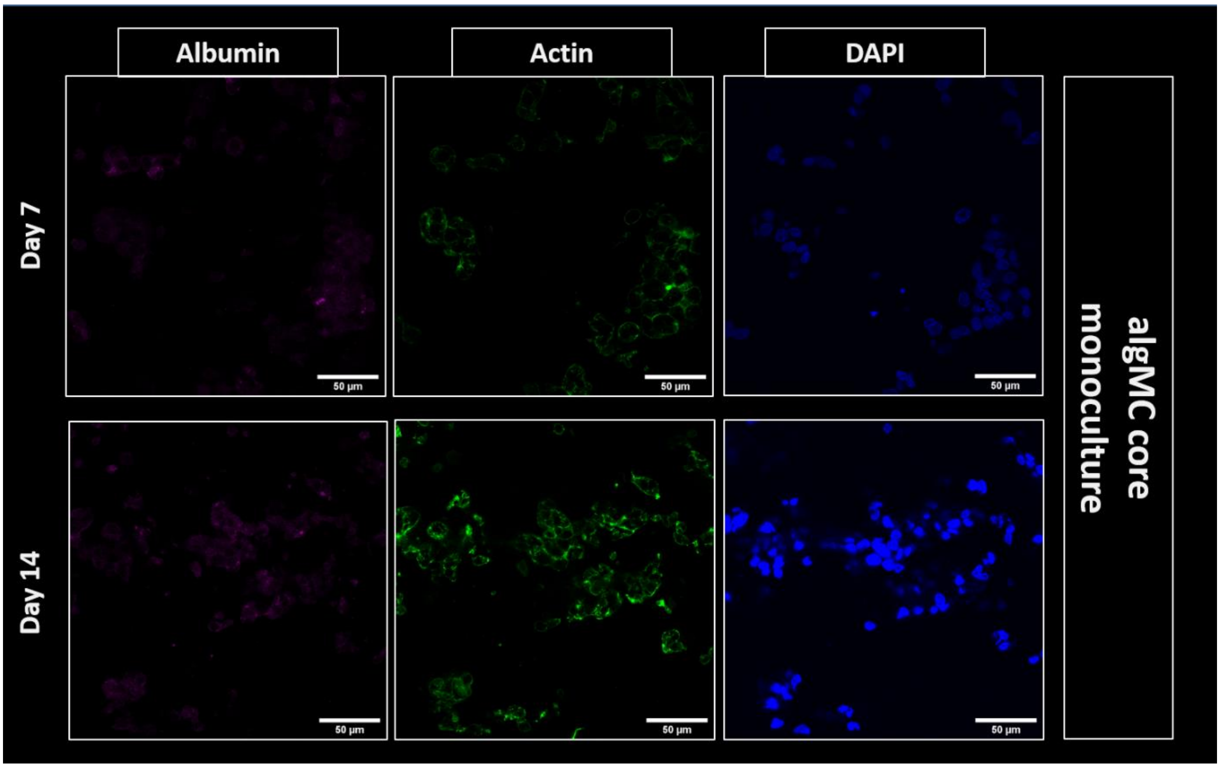

Supplementary figure S3: Cluster formation and biomarker expression of HepG2 cells embedded in shell compartment in monoculture (cell-free algMC core) at day 7 and 14 of cultivation. cLSM fluorescence images of HepG2 stained for Albumin (purple), nuclei (blue) and cytoskeletons (green); scale bars represent 50 µm. Figure shows the lower expression level of albumin in monoculture condition in comparison to co-culture conditions in the presence of fibrin- and plasma-supplemented core shown in Fig. 13A I.

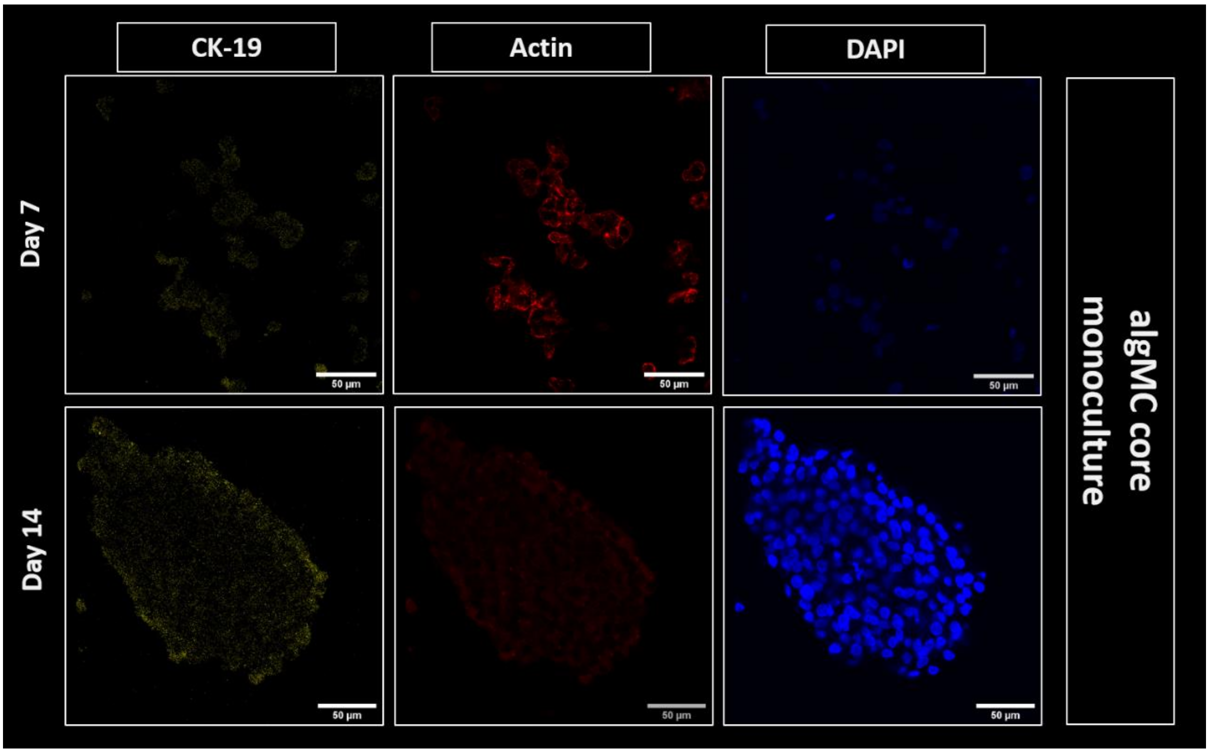

Supplementary figure S4: Cluster formation and biomarker expression of HepG2 cells embedded in shell compartment in monoculture (cell-free algMC core) at day 7, and 14 of cultivation. cLSM fluorescence image of HepG2 stained for CK-19 (yellow), nuclei (blue) and cytoskeletons (red); scale bars 50 µm. Figure shows the lower expression level of CK-19 in monoculture condition in comparison to co-culture conditions in presence of fibrin and plasma supplemented cores shown in Fig. 13B I.

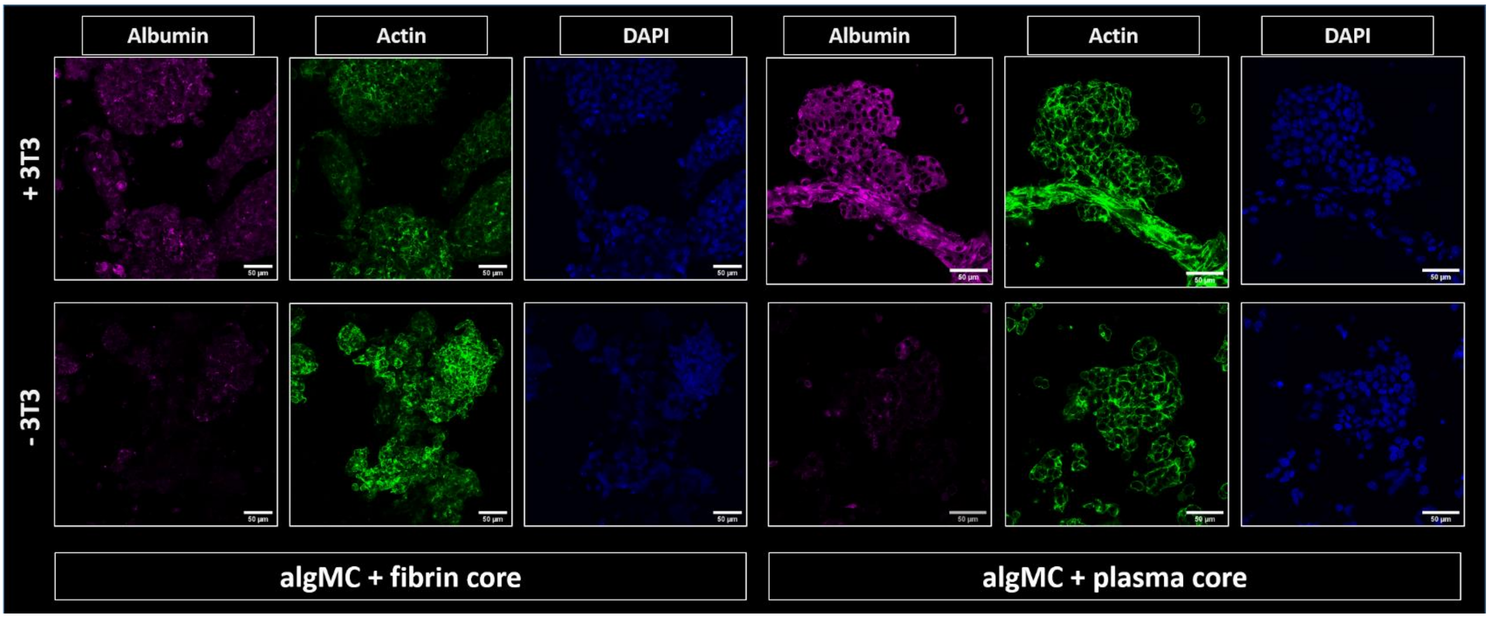

Supplementary figure S5: Albumin expression of HepG2 clusters formed in shell compartment in co-culture with NIH 3T3 embedded in core compartment (upper panel) and in monoculture (cell-free core; lower panel). In both, co-culture and monoculture, the core was composed of algMC + fibrin (left) and algMC + plasma (right). Confocal images of HepG2 stained for Albumin (purple), nuclei (blue) and cytoskeletons (green); scale bar represents 50 µm. Images also clearly show the influence of co-cultured NIH 3T3 cells in enhancing the albumin expression levels of hepatocytes in comparison to monoculture conditions.

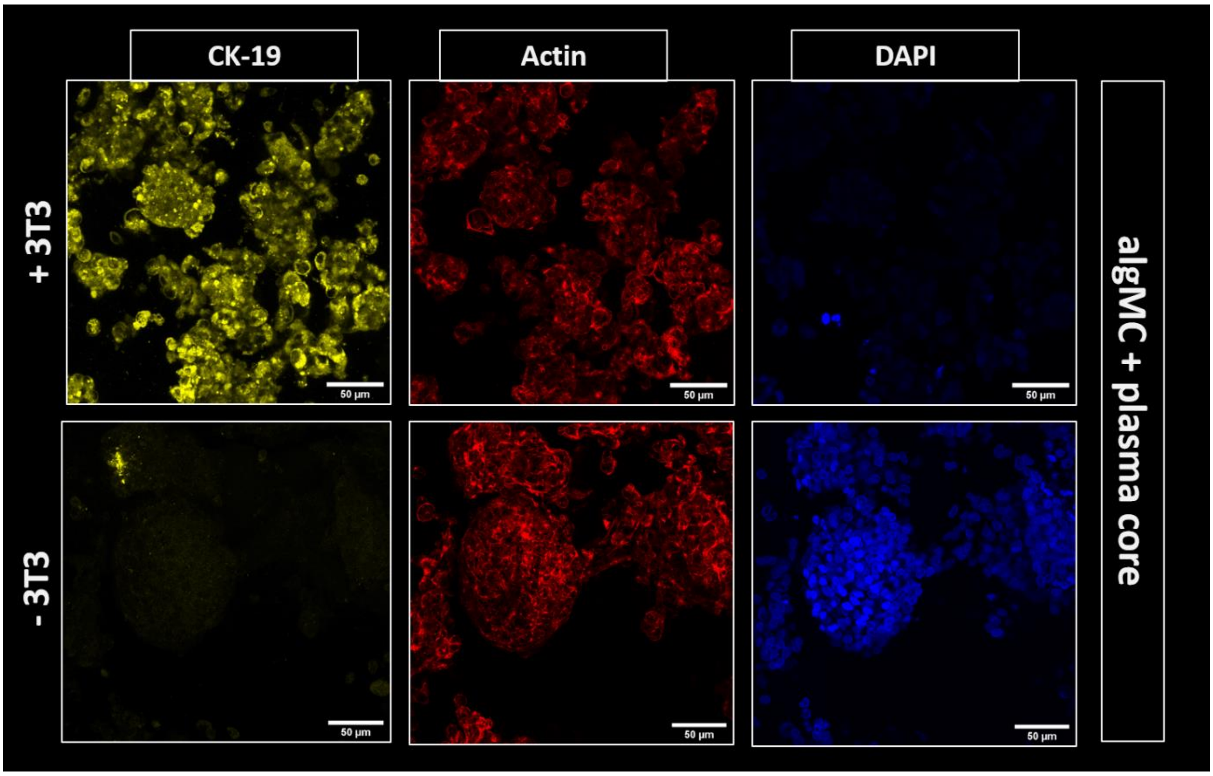

Supplementary figure S6: CK-19 expression of HepG2 clusters formed in shell compartment in co-culture with NIH 3T3 cells embedded in core compartment (upper panel) and in monoculture (cell-free core; lower panel). In both co-culture and monoculture, the core was composed of algMC + plasma. Confocal images of HepG2 stained for CK-19 (yellow), nuclei (blue) and cytoskeletons (red); scale bar = 50 µm. Images also clearly show the influence of co-cultured NIH 3T3 in enhancing the CK-19 expression levels of hepatocytes in comparison to monoculture conditions appearing in the higher intensity image of CK-19 in the upper panel.

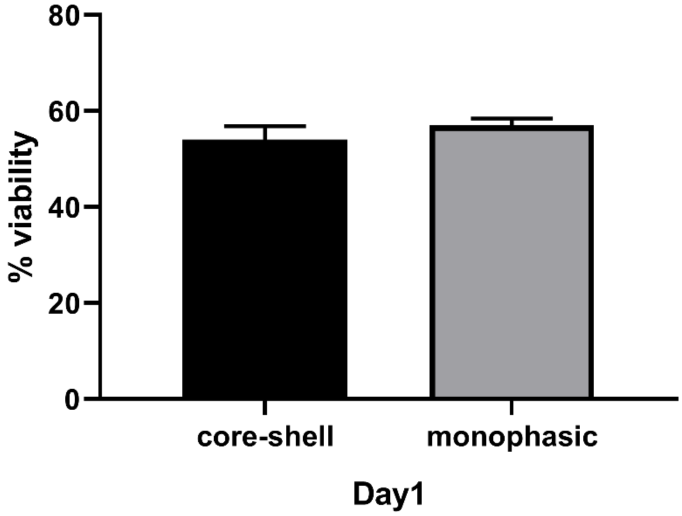

Supplementary figure S7. Viability ratio of HepG2 cells embedded in algMC+matrigel printed as core-shell or monophasic scaffolds. Viability was assessed at day 1 post-printing. (n=6, mean±SD).
